# The *Plasmodium falciparum* histone methyltransferase SET10 participates in a chromatin modulation network crucial for intraerythrocytic development

**DOI:** 10.1128/msphere.00495-24

**Published:** 2024-10-24

**Authors:** Jean-Pierre Musabyimana, Sherihan Musa, Janice Manti, Ute Distler, Stefan Tenzer, Che Julius Ngwa, Gabriele Pradel

**Affiliations:** 1 Division of Cellular and Applied Infection Biology, RWTH Aachen University, Aachen, Germany; 2 Institute of Immunology, University Medical Centre of the Johannes-Gutenberg University, Mainz, Germany; University of Georgia, Athens, Georgia, USA

**Keywords:** malaria, histone methyltransferase, transcription, DNA replication, epigenetic gene regulation, chromatin modulation, intraerythrocytic replication

## Abstract

The lifecycle progression of the malaria parasite *Plasmodium falciparum* requires precise tuning of gene expression including histone methylation. The histone methyltransferase *Pf*SET10 was previously described as an H3K4 methyltransferase involved in *var* gene regulation, making it a prominent antimalarial target. In this study, we investigated the role of *Pf*SET10 in the blood stages of *P. falciparum* in more detail, using tagged *Pf*SET10-knockout (KO) and -knockdown (KD) lines. We demonstrate a nuclear localization of *Pf*SET10 with peak protein levels in schizonts. *Pf*SET10 deficiency reduces intraerythrocytic growth but has no effect on gametocyte commitment and maturation. Screening of the *Pf*SET10-KO line for histone methylation variations reveals that lack of *Pf*SET10 renders the parasites unable to mark H3K18me1, while no reduction in the H3K4 methylation status could be observed. Comparative transcriptomic profiling of *Pf*SET10-KO schizonts shows an upregulation of transcripts particularly encoding proteins linked to red blood cell remodeling and antigenic variation, suggesting a repressive function of the histone methylation mark. TurboID coupled with mass spectrometry further highlights an extensive nuclear *Pf*SET10 interaction network with roles in transcriptional regulation and mRNA processing, DNA replication and repair, and chromatin remodeling. The main interactors of *Pf*SET10 include ApiAP2 transcription factors, epigenetic regulators like *Pf*HDAC1, chromatin modulators like *Pf*MORC and *Pf*ISWI, mediators of RNA polymerase II, and DNA replication licensing factors. The combined data pinpoint *Pf*SET10 as a histone methyltransferase essential for H3K18 methylation that regulates nucleic acid metabolic processes in the *P. falciparum* blood stages as part of a comprehensive chromatin modulation network.

**IMPORTANCE:**

The fine-tuned regulation of DNA replication and transcription is particularly crucial for the rapidly multiplying blood stages of malaria parasites and proteins involved in these processes represent important drug targets. This study demonstrates that contrary to previous reports the histone methyltransferase *Pf*SET10 of the malaria parasite *Plasmodium falciparum* promotes the methylation of histone 3 at lysine K18, a histone mark to date not well understood. Deficiency of *Pf*SET10 due to genetic knockout affects genes involved in intraerythrocytic development. Furthermore, in the nuclei of blood-stage parasites, *Pf*SET10 interacts with various protein complexes crucial for DNA replication, remodeling, and repair, as well as for transcriptional regulation and mRNA processing. In summary, this study highlights *Pf*SET10 as a methyltransferase affecting H3K18 methylation with critical functions in chromatin maintenance during the development of *P. falciparum* in red blood cells.

## INTRODUCTION

Understanding the epigenetic mechanisms of gene regulation is critical for identifying drivers of disease as a first step toward therapies targeting transcriptional mechanisms of disease onset. The use of anti-epigenetic agents is among others being explored for cancer and pain therapy with the first epigenome-targeting drugs having received approval (reviewed in references 1–4). The importance of epigenetics has also been acknowledged as a main driver of the lifecycle progression of malaria parasites, which are responsible for 249 million infections and 608,000 deaths per year (5). When the 23 Mb genome of *Plasmodium falciparum*, the causative agent of malaria tropica, was sequenced in 2002, roughly 5,300 genes coding for core proteins including a varying number of subtelomeric multigene families like *var*, *rif*, *stevor,* and *pfmc-2tm* were identified (6). Subsequent proteomic and transcriptomic analyses demonstrated lifecycle stage specificity for the majority of the gene products (7–11) and permitted a first glimpse into the tightly regulated transcriptional program that is based on well-coordinated sequences of gene activation and silencing needed by the parasite to progress from one lifecycle stage to another.

A significant part of epigenetic control in *Plasmodium* involves the posttranslational modification (PTM) of its histones, for example, *via* histone acetylation and methylation (reviewed in references 12, 13). Histone PTMs in malaria parasites have particularly been studied during expression of the 60 *var* genes encoding the *P. falciparum* erythrocyte membrane protein *Pf*EMP1 (14–16; reviewed in references 12, 17–20). The switch of *var* gene expression and thus *Pf*EMP1 structure alters the antigenic type of the infected red blood cells (RBCs) and in consequence pathogenesis of malaria. Only the active *var* gene copy exhibits a euchromatic state characterized by the two histone PTMs H3K9ac and H3K4me3, while *var* gene silencing is linked to H3K9me3 and H3K36me3 and the binding of heterochromatin protein HP1 (14, 15, 21–28). Overarching regulatory structures like reader complexes or nucleosome composition modifications support *var*-regulating PTMs (29, 30). Additional factors regulating the monoallelic expression of *var* genes include long non-coding RNAs (31, 32).

The genome of *P. falciparum* encodes 10 SET [Su(var)3–9-'Enhancer of zeste-Trithorax]-domain-containing lysine-specific histone methyltransferases (HMTs), termed *Pf*SET1 to *Pf*SET10, which are abundantly transcribed in the asexual and sexual blood stages (33–35). Treatment of blood-stage parasites with the commercially available inhibitor BIX-01294, known to target G9a lysine-specific HMTs, resulted in impaired intraerythrocytic replication, gametocyte development, and gametogenesis (36). Comparative transcriptomics between BIX-01294-treated and untreated immature, mature, and activated gametocytes further demonstrated the deregulation of various genes, particularly affecting antigenic variation, translation, and host cell remodeling.

To date, the *Pf*SET proteins have not been investigated in detail. Previous studies indicated that *Pf*SET2 and *Pf*SET10 are important for immune evasion of *P. falciparum*. While *Pf*SET2 (also termed *Pf*SETvs) is responsible for H3K36me3 marks needed to repress *var* gene expression (26), *Pf*SET10 was described as a H3K4 methyltransferase with suggested essential functions in maintaining the active *var* gene in a poised state during cellular division by marking of H3K4me2 and H3K4me3 (35; reviewed in reference 37). However, a subsequent report demonstrated that *Pf*SET10 deficiency had no significant effect either on intraerythrocytic replication or on *var* gene expression (38). In accordance with these findings, a recent independent study confirmed that *Pf*SET10 is not required for mutually exclusive *var* gene expression and switching (39).

Here, we investigated the role of *Pf*SET10 for the *P. falciparum* blood stages in further detail. We demonstrate that *Pf*SET10 is a methyltransferase essential for H3K18, but not H3K4 methylation. Lack of *Pf*SET10 leads to an upregulation of genes linked to RBC remodeling and antigenic variation. Furthermore, *Pf*SET10 forms extensive networks with proteins important for DNA replication, RNA synthesis, and chromatin modulation.

## RESULTS

### *Pf*SET10 localizes to the nuclei of the *P. falciparum* blood stages

**Fig 1:**
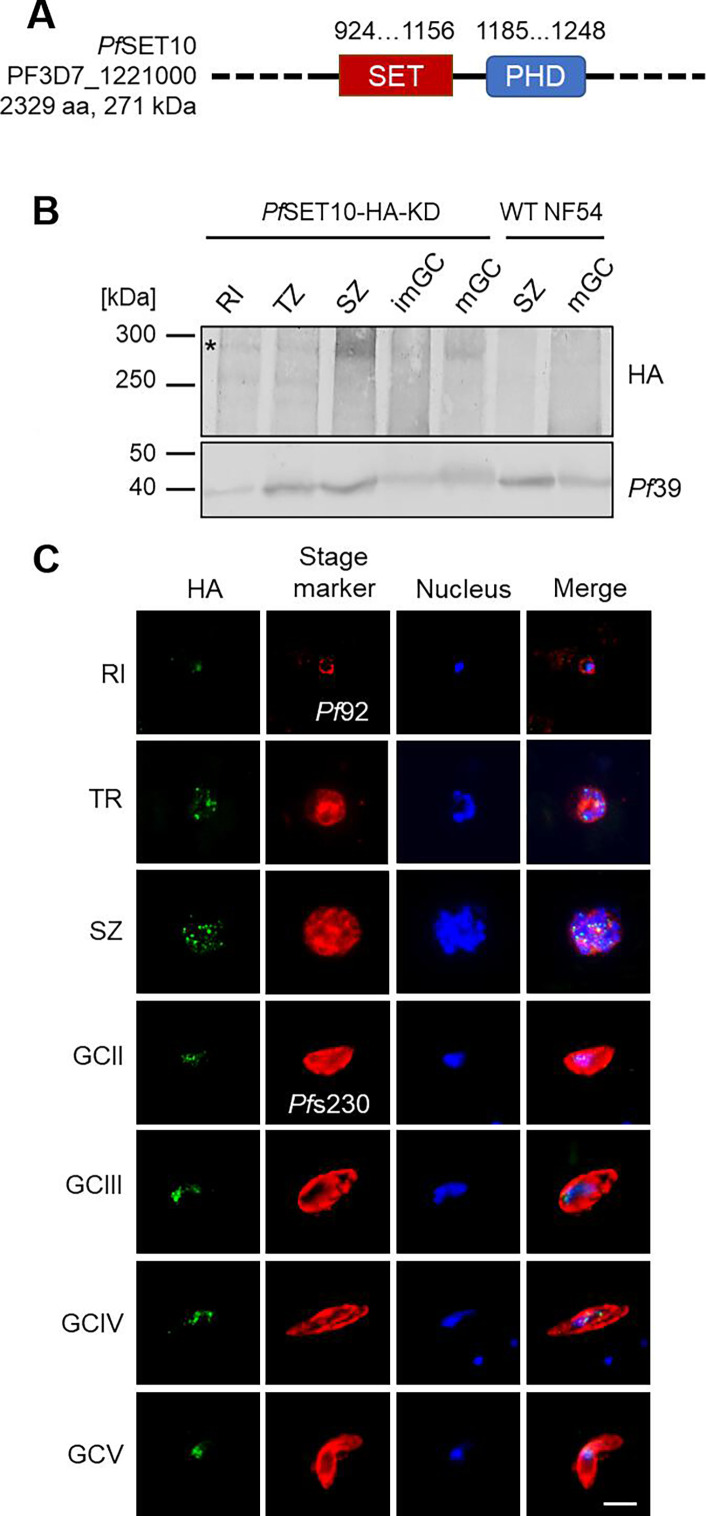
*Pf*SET10 is expressed in the *P. falciparum* blood stages. (**A**) Schematic depicting *Pf*SET10. The positions of the SET and PHD domains are indicated. (**B**) Protein expression of *Pf*SET10-HA in blood-stage parasites. Western blot analysis of lysates from rings (RI), trophozoites (TZ), schizonts (SZ), and immature and mature gametocytes (imGC, mGC) of line *Pf*SET10-HA-KD was employed, using rat anti-HA antibody to detect *Pf*SET10-HA (~275 kDa; asterisk). As a negative control, WT NF54 lysate was used. Immunoblotting with rabbit antisera against *Pf*39 (~39 kDa) served as loading control. (**C**) Localization of *Pf*SET10-HA in blood stage nuclei. Methanol-fixed RI, TZ, SZ, and GC stages II-V of line *Pf*SET10-HA-KD were immunolabeled with rat anti-HA antibody (green). Asexual blood stages and gametocytes were highlighted with rabbit antisera directed against *Pf*92 and *Pf*s230, respectively (red); nuclei were highlighted with Hoechst 33342 nuclear stain (blue). Bar, 5 µm.

*Pf*SET10 is a 271-kDa protein (PF3D7_1221000; 2,329 aa) comprising a central SET and PHD zinc finger domain (Fig. 1A). For functional characterization, we generated a conditional *Pf*SET10-HA-KD line (using the pSLI-HA-*glmS* vector) by fusing the 3′-region of *pfset10* with the sequences coding for a hemagglutinin A (HA) tag and for the *glmS* element (Fig. S1A) (40, 41). Vector integration was confirmed by diagnostic PCR (Fig. S1A and B). To prove the conditional downregulation of *Pf*SET10-HA synthesis, asexual blood stage parasites of line *Pf*SET10-HA-KD were treated with glucosamine hydrochloride (GlcN) for 72 h, and lysates were subjected to Western blotting. In untreated cultures, a prominent *Pf*SET10-HA band was detected at ~275 kDa. Upon the addition of GlcN, *Pf*SET10-HA levels were significantly reduced to 68.6% ± 19.91% (2.5 mM) and 15.8% ± 7.51% (5 mM), respectively, compared to the untreated *Pf*SET10-HA-KD control, as shown by quantitative Western blotting (Fig. S2A and B).

The untreated *Pf*SET10-HA-KD line was initially used for protein expression analyses. Western blotting using anti-HA antibody demonstrated the presence of *Pf*SET10-HA in the asexual blood stages and immature and mature gametocytes, whereas no protein band was detected in the WT NF54 (Fig. 1B). *Pf*SET10-HA bands exhibited the expected molecular weight (~275 kDa) with particular high levels in schizonts; in some occasions, smaller protein bands (~260 kDa) could additionally be detected. Indirect immunofluorescence assays further assigned *Pf*SET10 to the parasites’ nuclei (Fig. 1C). In the asexual blood stages, *Pf*SET10-HA localized to unique spots at the nuclear periphery, while in gametocytes broader, *Pf*SET10-positive areas were observed. No labeling was detectable in the WT NF54 blood stages (Fig. S3A).

### *Pf*SET10 deficiency reduces intraerythrocytic growth but does not affect gametocyte formation

We evaluated the effect of *Pf*SET10 deficiency on intraerythrocytic growth and gametocyte development, using an established *Pf*SET10-KO line, which was previously generated via selection-linked integration-mediated targeted gene disruption, using vector pSLI-TGD (38, 42). The transgenic parasites express a truncated N-terminal fragment of *Pf*SET10 fused with green fluorescent protein (GFP), which lacks the SET and PHD domains. We previously showed that the asexual blood stage parasites of the *Pf*SET10-KO line displayed normal morphologies (38); in addition, no morphological differences were observed in *Pf*SET10-KO gametocytes (Fig. S3B). In conformation with our previous reports, *Pf*SET10-KO parasites exhibited reduced parasitemia compared to WT NF54 with normal stage progression through the replication cycles (Fig. S4A and B). No significant differences in gametocyte development were observed between *Pf*SET10-KO and WT NF54 over a period of 13 days (Fig. S4C).

Intraerythrocytic development was also investigated in the *Pf*SET10-HA-KD line. Here, growth assays revealed no differences in parasitemia, when synchronized asexual blood stages of line *Pf*SET10-HA-KD were treated with 2.5 mM GlcN over a period of 96 h compared to untreated cultures; GlcN-treated and untreated WT NF54 parasites served as controls (Fig. S5A and B). Furthermore, no differences in gametocyte numbers were observed between line *Pf*SET10-HA-KD and WT NF54 (Fig. S5C). To investigate both gametocyte commitment and gametocyte maturation, we treated gametocytes of line *Pf*SET10-HA-KD with GlcN either prior to or after induction, and treatment was continued for 7 days, while untreated transgenic parasites and treated and untreated WT NF54 gametocytes served as controls.

### *Pf*SET10 deficiency abolishes H3K18 mono-methylation but not H3K4 methylation

**Fig 2:**
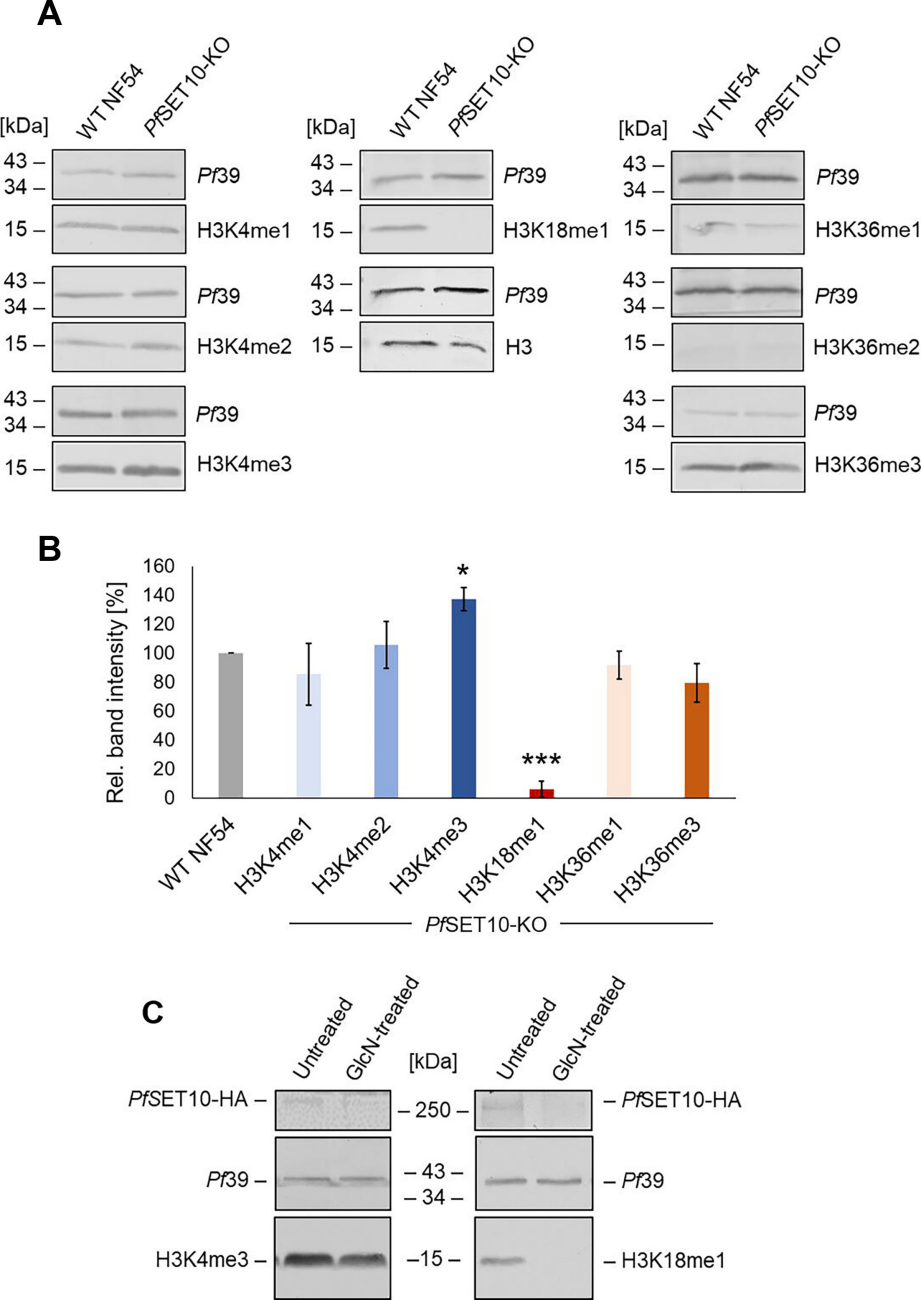
*Pf*SET10 deficiency abolishes H3K18me1 methylation. (**A**) Lysates of schizonts of line *Pf*SET10-KO and WT NF54 were immunoblotted with rabbit antibodies directed against selected histone 3 (H3) lysine (K) methylation marks as indicated (~15 kDa). Rabbit antisera directed against H3 and *Pf*39 (~39 kDa) were used as loading controls. (**B**) Quantification of H3K methylation. Western blots were performed, using rabbit antibodies as described in panel **A**. The respective H3K methylation signals were evaluated by measuring the band intensities of three independent immunoblots using Image J; the values were normalized to the respective *Pf*39 protein band. Results are shown as mean ± SD (WT NF54 set to 100% for each mark). Significant differences in H3 methylation levels between *Pf*SET10-KO and WT NF54 are indicated (*P ≤ 0.05; ***P ≤ 0.001; Student’s *t*-test). (**C**) Asexual blood stage parasites of line *Pf*SET10-HA-KD were treated or not with 2.5 mM GlcN and the lysates were immunoblotted with antibodies directed against H3K4me3 and H3K18me1 to detect the respective methylation mark (~15 kDa). *Pf*SET10-HA (~275 kDa) was detected with anti-HA antibodies; immunoblotting with rabbit antisera directed against *Pf*39 (~39 kDa) was used as a loading control.

To determine the methylation activity of *Pf*SET10, we investigated the effect of *Pf*SET10 deficiency on seven histone 3 (H3) lysine (K) methylation marks, for which commercial antibodies were available, that is, H3K4me1, H3K4me2, H3K4me3, H3K36me1, H3K36me2, H3K36me3, and H3K18me1. Lysates generated from schizonts of line *Pf*SET10-KO and WT NF54 were immunoblotted with rabbit antibodies directed against the above H3K methylation marks. No reductions in band intensities were identified in the *Pf*SET10-KO line compared to WT NF54 for any methylation mark concerning H3K4 and H3K36 (Fig. 2A). Generally, only weak signals were detected for H3K36me2, indicating that this mark is not relevant in schizonts. By contrast, no signal was detected for the methylation mark H3K18me1 in the *Pf*SET10-KO line, whereas strong methylation bands were observed in WT NF54 (Fig. 2A). Immunoblotting with antibodies directed against histone H3 and *Pf*39 served as loading controls in the experiments.

The methylation levels were subsequently evaluated via quantitative Western blotting. Methylation band signals from three independent Western blots were quantified for each mark, normalized to the respective *Pf*39 levels and compared between *Pf*SET10-KO and WT NF54 (Fig. 2B). Signal quantification confirmed a significant reduction of H3K18me1 methylation to 6.1% ± 5.58% in the *Pf*SET10-KO. In addition, increased H3K4me2 and H3K4me3 levels (105.9% ± 16.18% and 137.3% ± 8.15%, respectively) could be detected in *Pf*SET10-KO schizonts (Fig. 2B).

The methylation effects of *Pf*SET10 on H3K4me3 and H3K18me1 were confirmed using line *Pf*SET10-HA-KD. Asexual blood stages of line *Pf*SET10-HA-KD were treated with 2.5 mM GlcN for 72 h. Lysates were immunoblotted with anti-HA antibodies to highlight *Pf*SET10-HA in the samples, while the H3K4me3 and H3K18me1 methylation marks were detected with the respective antibodies. Treatment of line *Pf*SET10-HA-KD with GlcN reduced the *Pf*SET10-HA levels, as expected, and further abolished the H3K18me1 mark, while the H3K4me3 signal did not alter (Fig. 2C). The levels of *Pf*39, used for loading and viability control, were not affected by the GlcN treatment.

**Fig 3:**
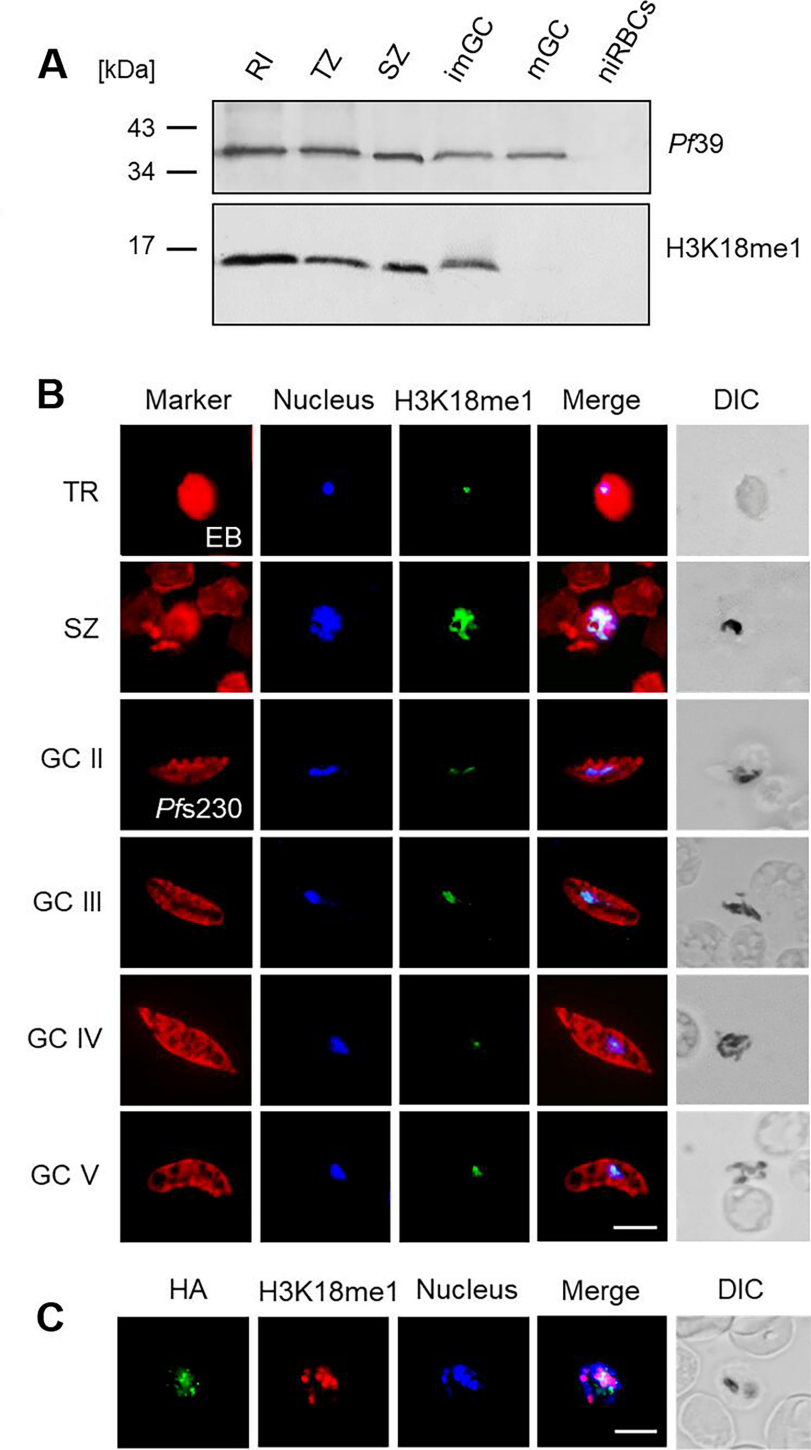
The H3K18me1 methylation mark associates with blood-stage nuclei. (**A**) H3K18me1 levels in the parasite blood stages. Lysates of WT NF54 ring (RI), trophozoites (TZ), schizonts (SZ), and immature and mature gametocytes (imGC, mGC) were immunoblotted with rabbit anti-H3K18me1 antibodies to detect the methylation mark (~15 kDa). Immunoblotting with rabbit antisera directed against *Pf*39 (~39 kDa) was used as a loading control. (**B**) Localization of H3K18me1 in blood-stage nuclei. Methanol-fixed rings RI, TZ, SZ, and GC stages II-V of WT NF54 were immunolabeled with rabbit anti-H3K18me1 antibody (green). Asexual blood stages and gametocytes were highlighted with Evans blue and mouse anti-*Pf*s230 antisera, respectively (red); nuclei were highlighted with Hoechst 33342 nuclear stain (blue). (**C**) The H3K18me1 mark colocalizes with *Pf*SET10. Schizonts of line *Pf*SETS10-HA-KD were immunolabeled as described in panel **B**, using an anti-H3K18me1 antibody (red), while rat anti-HA antibody was used to detect *Pf*SET10-HA (green). DIC, differential interference contrast. Bar, 5 µm.

We then investigated the H3K18me1 mark in the asexual and sexual blood stages during stage development. Western blotting demonstrated particularly prominent signals in the asexual blood stages, while the methylation mark vanished in the maturing gametocytes (Fig. 3A). Immunofluorescence assays further assigned the H3K18me1 mark to the blood stage nuclei with strong signals in schizonts (Fig. 3B). Furthermore, the *Pf*SET10-HA signal partially overlapped with the H3K18me1 methylation mark, when the *Pf*SET10-HA-KD line was subjected to immunofluorescence assays (Fig. 3C).

### *Pf*SET10 deficiency results in the upregulation of genes linked to antigenic variation and RBC remodeling

**Fig 4:**
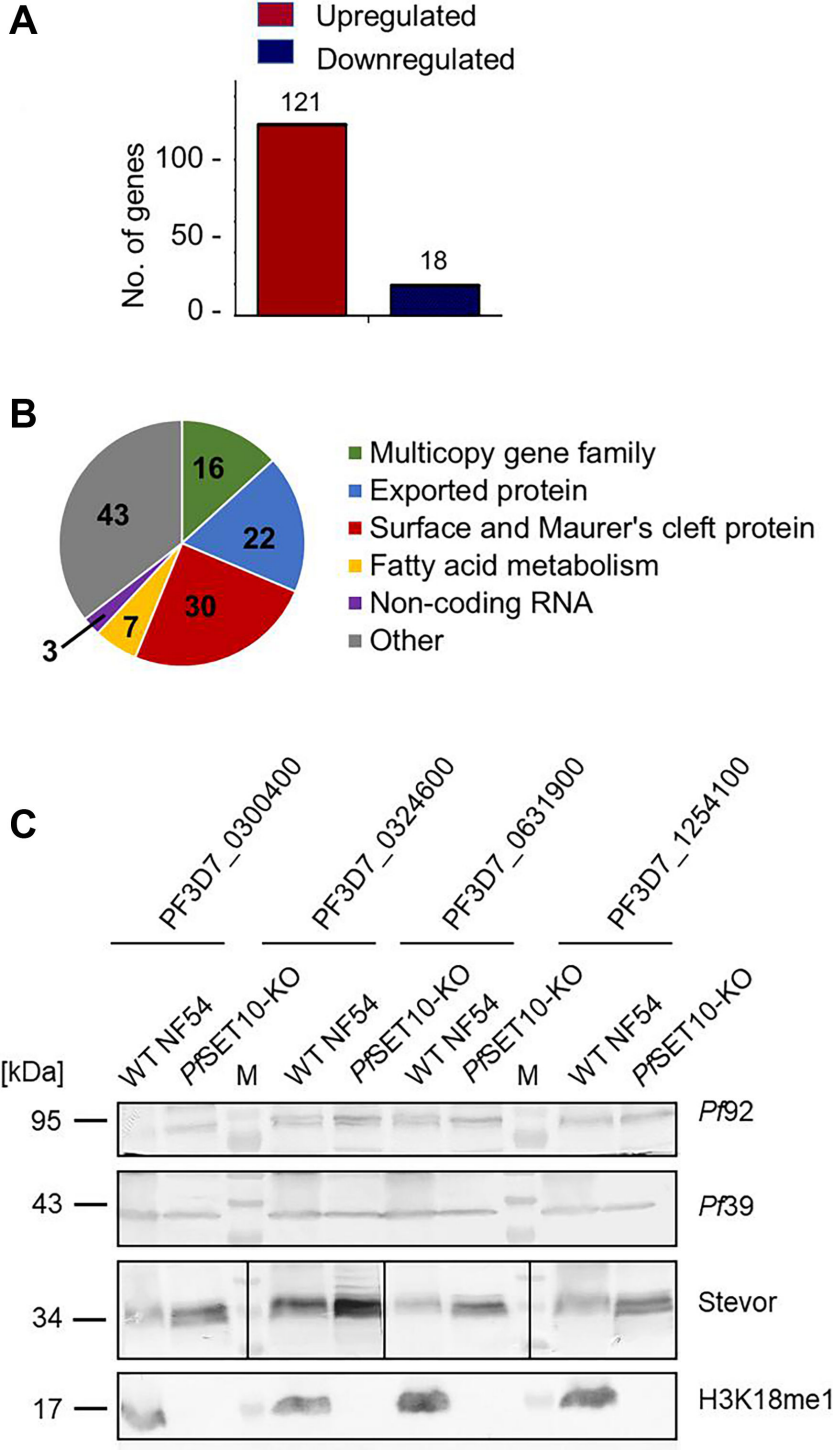
*Pf*SET10 deficiency leads to the upregulation of genes linked to antigenic variation and RBC remodeling. (**A**) Numbers of transcriptionally deregulated genes in line *Pf*SET10-KO. RNA-seq-based comparative transcriptomics analyses of transcripts from schizonts of line *Pf*SET10-KO and WT NF54 identified 139 deregulated genes. (**B**) Pie chart depicting the numbers of upregulated genes in line *Pf*SET10-KO grouped by function. (**C**) *Pf*SET10 deficiency leads to increased STEVOR levels. Lysates of asexual blood stage parasites of line *Pf*SET10-KO and WT NF54 were immunoblotted with rabbit antibodies directed against selected STEVOR proteins (~35 kDa; indicated by gene ID). Rabbit antisera directed against *Pf*92 (~92 kDa) and *Pf*39 (~39 kDa) were used as loading and stage controls; rabbit anti-H3K18me1 antibodies highlight the methylation mark (~15 kDa). M, molecular weight marker.

We carried out a comparative transcriptomics analysis using RNA isolated from schizonts of line *Pf*SET10-KO and WT NF54. A total of 139 deregulated genes were identified by RNA-seq. The majority (121 genes) were transcriptionally upregulated in the *Pf*SET10-KO (log2 fold changes between 1.3 and 5.9), while 18 genes were transcriptionally downregulated (log2 fold changes between −1.4 and −8.4; Fig. 4A; Table S1). Genes that were transcriptionally upregulated had peak expression assigned to the ring and ookinete stages, whereas downregulated genes exhibited peak expression in rings and trophozoites (Fig. S6A). Upregulated RNAs included those coding for members of the *var*, *rif*, and *stevor* multigene families, proteins of the infected RBC membrane and the Maurer’s clefts like CLAG3.2, SBP1, HRP, RESA, RESA3, REX1, MAHRP2, and MaTrA, exported proteins, for example, PHIST family members, and lysophospholipase LPL120 and three acyl-CoA synthetases; further three non-coding RNAs were identified (Fig. 4B; Table S1). Downregulated genes included mainly those coding for chaperones and ribosome units. In accordance with these data, gene ontology (GO) enrichment analysis assigned the transcriptionally upregulated genes to biological processes such as cytoadherence, cell adhesion, and erythrocyte aggregation with molecular functions in cell adhesion molecule and host cell surface binding. Further molecular functions included coenzyme A ligase and fatty acid ligase activities (Fig. S6B).

We subsequently investigated the impact of *Pf*SET10 deficiency on the protein synthesis of four STEVOR proteins (PF3D7_0300400, PF_0324600, PF3D7_0631900, and PF3D7_1254100), which were transcriptionally upregulated in the comparative RNA-seq analysis. Lysates of *Pf*SET10-KO and WT NF54 schizonts were subjected to Western blotting, using rabbit antibodies directed against the four STEVOR proteins, while rabbit antibodies directed against *Pf*92 and *Pf*39 served as stage and loading control, respectively (Fig. 4C). Immunoblotting detected increased band intensities for all of the STEVOR proteins in the absence of *Pf*SET10. Immunoblotting with anti-H3K18me1 antibodies confirmed the absence of this methylation mark in the *Pf*SET10-KO line.

### The *Pf*SET10 interactome comprises proteins of DNA and RNA metabolic processes

To determine the *Pf*SET10 interaction network, we generated a transgenic line that endogenously expresses *Pf*SET10 fused with the enhanced TurboID version of the *Escherichia coli* biotin ligase BirA in addition to GFP, using vector pSLI-TurboID-GFP (Fig. S7A) (43, 44). Successful vector integration was demonstrated by diagnostic PCR (Fig. S7B).

Western blot analyses of asexual blood stage lysates prepared from the transgenic line, using mouse anti-GFP antibody, demonstrated the expression of *Pf*SET10-TurboID-GFP with a molecular weight of ~300 kDa (Fig. S7C). No protein band was detected in lysates of non-infected RBCs; immunoblotting with anti-*Pf*39 served as loading control. Indirect immunofluorescence analyses, using anti-GFP antibodies, confirmed the expression of *Pf*SET10-TurboID-GFP in the transgenic blood stage parasites and demonstrated the nuclear localization of the fusion protein (Fig. S7D).

Subsequent Western blotting was employed to confirm protein biotinylation in the transgenic line. Asexual blood stages of line *Pf*SET10-TurboID-GFP were treated with 50 µM biotin for 10 min. Immunoblotting of the respective lysates using streptavidin conjugated to alkaline phosphatase detected multiple protein bands indicative of biotinylated proteins, including a protein band of ~300 kDa, likely representing biotinylated *Pf*SET10-TurboID-GFP (Fig. S8A). In lysates of transgenic parasites that were not treated with biotin, only weak protein bands were detected, indicative of endogenous biotin, and no biotin-positive protein bands were detected in the biotin-treated WT NF54. Immunofluorescence assays, using fluorophore-conjugated streptavidin, confirmed the presence of biotinylated proteins in the nuclei of schizonts and the signal overlapped with the *Pf*SET10-TurboID-GFP signal, while no biotinylated proteins were detected in biotin-treated WT NF54 parasites (Fig. S8B).

**Fig 5:**
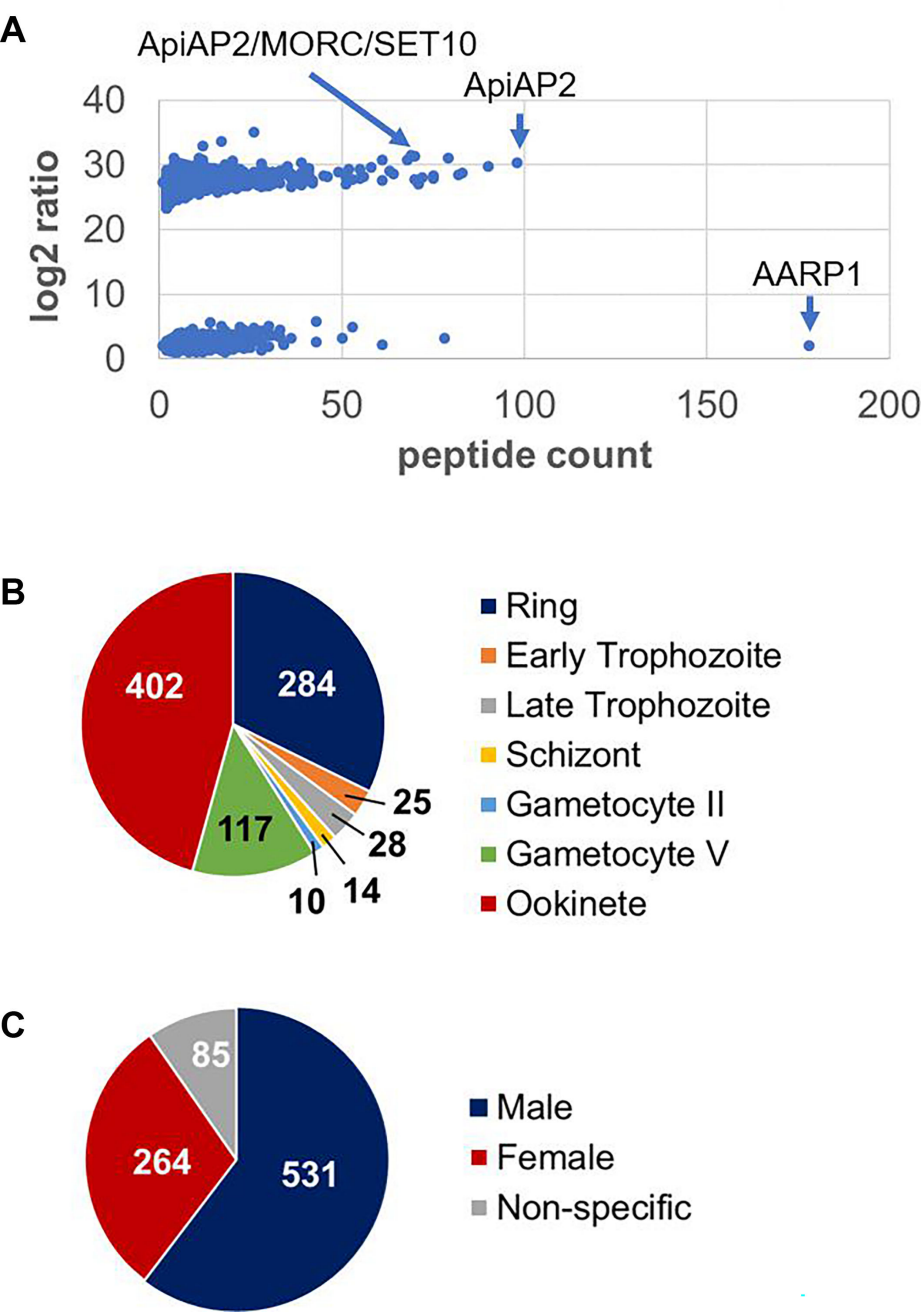
TurboID analysis identifies *Pf*SET10 interactors in blood-stage parasites. (**A**) Scatter diagram depicting total *Pf*SET10 interactors. TurboID analysis using schizonts of line *Pf*SET10-TurboID-GFP resulted in the identification of 1,204 hits, which were plotted by peptide count versus log2 ratio. Selected hits are highlighted. (**B**) Pie chart depicting *Pf*SET10 interactors (log2 ratio ≥ 23) grouped by peak transcript expression in seven asexual and sexual lifecycle stages (PlasmoDB). (**C**) Pie chart depicting *Pf*SET10 interactors (log2 ratio ≥ 23) grouped by male versus female-enriched transcript expression (PlasmoDB).

The biotinylated proteins were purified from schizonts of line *Pf*SET10-TurboID-GFP via streptavidin-coated beads; WT NF54 lysates served as control. The biotinylated proteins were then prepared for mass spectrometry to identify potential *Pf*SET10 interactors. A total of 1,204 hits were identified (Tables S2 and S3). When we plotted peptide count numbers versus log2 fold change, two distinct populations of proteins could be distinguished (Fig. 5A). While the first group exhibited a log2 fold ratio below 6 and included, for example, various ribosomal and proteasomal proteins as well as the 646 kDa protein asparagine and aspartate-rich protein 1 (AARP1), the second group with a log2 ratio above 23 comprised mostly proteins exclusively assigned to the nucleus, like transcription factors, ApiAP2 proteins, epigenetic regulators, and chromatin-remodeling proteins like *Pf*MORC.

For further analyses, we focused on members of the second group (log2 ratio ˃ 23) and excluded proteins with predicted signal peptides (Fig. S8C; Table S3), resulting in 880 hits. The proteins exhibited peak expression profiles assigned to ookinetes and mature gametocytes as well as to ring stages (Fig. 5B). Noteworthy, the majority of hits had a predicted enrichment in male gametocytes (Fig. 5C). Furthermore, GO term analysis highlighted the involvement of *Pf*SET10 interactors in metabolic processes of nucleic acids and cellular macromolecules as well as of nitrogen compounds, while molecular functions that involve the potential *Pf*SET10 interactors comprise binding of DNA, RNA, and histones (Fig. S9).

The *Pf*SET10 interactors could be particularly assigned to three main functions, DNA replication, transcription, and mRNA processing (Table 1; Table S4). A total of 14 ApiAP2 transcription factors and seven other transcription factors were identified. In addition, 21 epigenetic regulators (including *Pf*SET10) were found. Furthermore, 17 RNA polymerases, 10 mediators of RNA polymerase II (MED proteins), 14 RNA helicases, and further proteins are related to transcription. In all, 18 small nuclear ribonucleoproteins and 29 mRNA splicing factors and associated proteins could furthermore be linked to mRNA processing. In addition, various proteins could be assigned to DNA replication, including 3 DNA polymerase subunits, 11 DNA helicases, 5 DNA replication licensing factors (MCM proteins), and 7 DNA repair-related proteins; further proteins have functions in, for example, DNA condensation and the regulation of replication (Table 1; Table S4). Noteworthy, we further identified chromatin remodeling proteins, which in addition to *Pf*MORC include *Pf*ISWI, *Pf*SWIP, *Pf*SNF2L, *Pf*CAF1A, and *Pf*CAF1B.

**TABLE 1:** Selected interactors of *Pf*SET10 with involvement in transcription, mRNA processing, and DNA replication

Function	Interactor
Transcription	
ApiAP2 transcription factors	e.g., AP2-G, AP2-G5, AP2-I, ApiAP2-L, AP2-LT, ApiAP2-O2, ApiAP2-O5, AP2-HC, AP2Tel, AP2-P, SIP2
Transcription factors, other	e.g., ADA2, MYB1, P44, TAF10, TFB2
Epigenetic regulators	e.g., BDP1-BDP7, PHD1, PHD2, HDA1, HDA2, HDAC1, HAT1, MORC, MYST, SET1-SET3, SET6
RNA polymerases	e.g., RNAPI, RPA2, RPB1-RPB6, RPB8-RPB10, RPC2, RPC40
Mediators of RNA POLII	MED4, MED6, MED7, MED8, MED14, MED17, MED18, MED21, MED22, MED31
RNA helicases	e.g., DBP1, DBP5, DBP10, DDX1, DDX17, DDX42, DHX57, DOZI, MTR4, UPF1, XPB, XPD
Others	e.g., FACT-L, ISWI, NIF3, NIF4, ORP2, SPT5, SPT6, SRCAP, SWIB
mRNA processing	
Small nuclear ribonucleoproteins	EFTUD2, LSM2, LSM3, LSM7, PRPF3, PRPF4, SART1, SMB1, SNP1, SNRPA, SNRPB2, SNRPC, SNRPD1-SNRPD3, SNRPG, SNRPF, SNRNP40
mRNA splicing factors	e.g., BUD31, BRR2, CEF1, CLF1, CWC15, CWF7, ISY1, PRP2, PRP16, PRP17, PRP22, PRP43, PRP45, PRP46, PRPF8, PRPF19, RBM22, SF1, SF3A1, SF3A2, SF3B2-SF3B4, SF3B6, XAB2
Others	e.g., AQR, CFIM25, THO2
DNA replication	
Apicomplexan kinetochore proteins	AKIT2, AKIT4, AKIT6
DNA repair proteins	MLH, MSH6, PMS1, RAD2, RAD50, RAD51, Rhp16
Origin of replication proteins	ORC2, ORC4, ORC5
DNA polymerases	Alpha subunit A, delta small subunit, epsilon subunit A
DNA replication licensing factors	MCM3, MCM6, MCM8, MCM9, MCMBP
DNA helicases, others	e.g., DDX3X, DNA2, FANCJ, PSH3, RUVB2, RUVB3, UvrD, WRN
Others	e.g., CAF1A, CAF1B, CDC6, CEN2, CRK4, FEN1, KIN17, RPA1, SNF2L, TOP3

**Fig 6:**
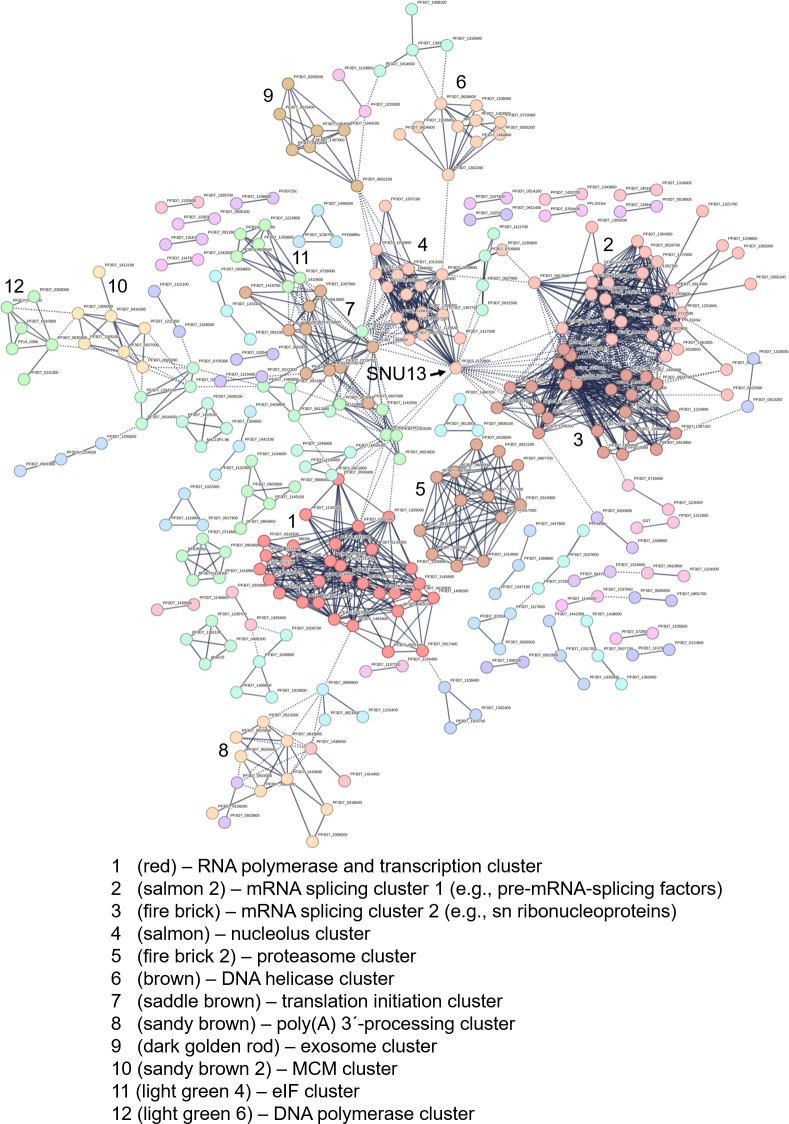
*Pf*SET10 interactors form a distinct network with nuclear proteins. A protein-protein network analysis of the *Pf*SET10 interactors in schizonts (log2 ratio ≥ 23; *n* = 880) was performed using STRING. The Markov clustering algorithm with a confidence level of 0.9 was applied; disconnected nodes were excluded. Clusters ≥ 6 proteins were considered. For a detailed view, see Table S5.

A STRING-based protein-protein interaction analysis was conducted, using the Markov clustering algorithm with a confidence level of 0.9. A total of 12 main clusters were identified (cluster size ≥ 6 proteins; Fig. 6; Table S5), which could be assigned to RNA polymerases and transcription (cluster 1), mRNA splicing and processing (clusters 2, 3, and 8), DNA replication (clusters 6, 10, and 12), the nucleolus (cluster 4), translation initiation (clusters 7 and 11), the proteasome (cluster 5), and the nuclear exosome (cluster 9). In the interaction network, particularly one protein played a key role by connecting clusters 2, 3, 4, and 7, that is, the small nuclear ribonucleoprotein SNU13 with predicted roles in mRNA splicing (Fig. 6).

### *Pf*SET10 interacts with *Pf*AP2-P, *Pf*ISWI, and *Pf*MORC as part of a chromatin modulation network

**Fig 7:**
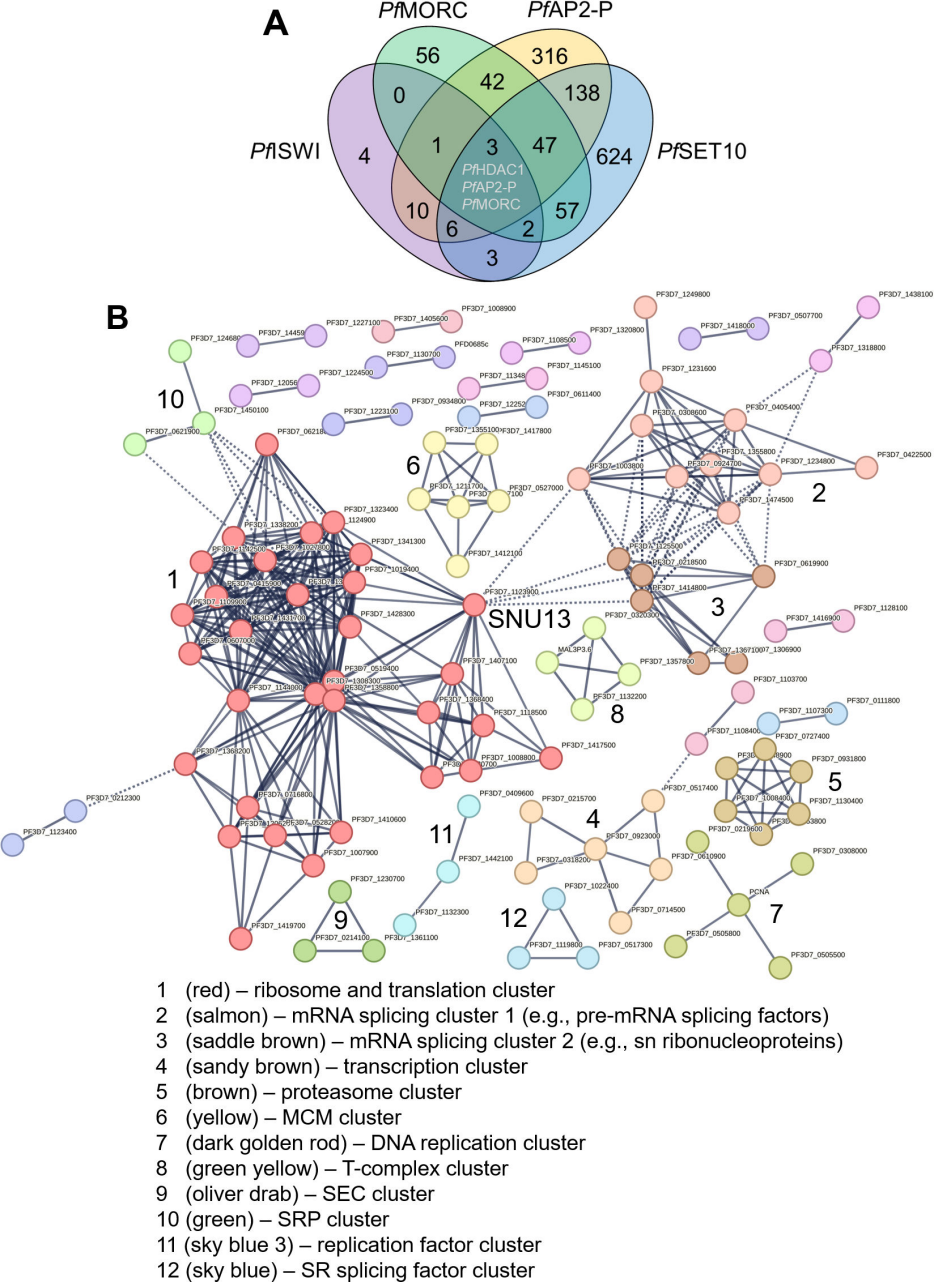
*Pf*SET10 forms a comprehensive interaction network with *Pf*AP2-P, *Pf*ISWI, and *Pf*MORC. (**A**) Venn diagram depicting interactors shared by *Pf*AP2-P, *Pf*ISWI, *Pf*MORC, and *Pf*SET10. The list of interactors was extracted from references 45–48. (**B**) Network analysis of the shared interactors. A protein-protein network analysis of the shared interactors (*n* = 309) was performed using STRING. The Markov clustering algorithm with a confidence level of 0.9 was applied; disconnected nodes were excluded. Clusters ≥ 3 proteins were considered. For a detailed view, see Table S6.

Among the top hits of nuclear interactors of *Pf*SET10 are three proteins recently identified as chromatin modulators, that is, the chromatin-associated microrchidia protein *Pf*MORC (interactor no. 2), ApiAP2 transcription factor *Pf*AP2-P (interactor no. 41), and the *var* gene promotor-associated chromatin remodeling protein *Pf*ISWI (interactor no. 46; see Table S3), for which protein interactomics data are available (45–48). We thus compared the interactors of *Pf*AP2-P, *Pf*ISWI, *Pf*MORC, and *Pf*SET10, and identified a total of 309 proteins, with which at least two of the bait proteins interacted (Fig. 7A; Table S6). The majority of interactions were found between *Pf*SET10 and *Pf*AP2-P as well as between *Pf*SET10 and PfMORC. One interactor was shared by all four bait proteins, that is, the histone deacetylase *Pf*HDAC1 (in addition to the bait proteins *Pf*MORC and *Pf*AP2-P).

The 309 interactors were subjected to STRING analysis, as described above, and 12 clusters (cluster size ≥ 3 proteins; Fig. 7B; Table S6) were identified. The majority of clusters were related to ribosomes and translation (cluster 1), transcription (cluster 4), mRNA processing (clusters 2, 3, and 12), and DNA replication (clusters 6, 7, and 11). Other clusters were assigned to the proteasome (cluster 5), the T-complex (cluster 8), SEC translocon proteins (cluster 9), and SRP (signal recognition particle) proteins (cluster 10). SNU13 formed a central protein with connections to clusters 1, 2, and 3.

## DISCUSSION

In *P. falciparum*, *Pf*SET10 has originally been designated a H3K4 methyltransferase crucial for maintaining the active *var* gene in a poised state during cellular division (35; reviewed in reference 37). In a later study, the ability to knock out the *pfset10* gene without altering *var* gene expression questioned the essentiality of *Pf*SET10 in *var* gene regulation (38). Here, we show that *Pf*SET10 is a methyltransferase essential for H3K18 but not H3K4 methylation. In accordance with previous reports (49, 50), this methylation mark is particularly present in the nuclei of schizonts but vanishes in gametocytes upon maturation. We further demonstrate that the lack of *Pf*SET10 completely abolishes the H3K18me1 mark in blood-stage parasites. *Pf*SET10 deficiency and hence the absence of H3K18me1 methylation in schizonts leads to the transcriptional upregulation of *rif*, *stevor,* and *var* genes as well as of genes encoding proteins involved in RBC remodeling, suggesting that *Pf*SET10 may be involved in gene repression during intraerythrocytic replication. Noteworthy in this context, a recent study by Wyss et al. (39) did not observe a significant deregulation of gene expression following *Pf*SET10 deficiency, hypothesizing that *Pf*SET10 is dispensable for the regulation of gene activities. While the H3K18me1 methylation mark to date is not well understood, it was found on the gene bodies of repressed genes of *Theileria* macroschizonts (51). In accord with our findings, peak H3K18me1 levels were observed in schizonts, whereas the methylation mark decreased during the differentiation of *T. annulata* from schizonts to merozoites, and chemical inhibition of the H3K18me1 methylation activity affected genes associated to macroschizont-to-merozoite transition.

A previous quantitative chromatin proteomics analysis revealed a highly dynamic histone PTM landscape in the blood stages of *P. falciparum* with euchromatic histone PTMs being abundant during schizogony and late gametocytes and heterochromatic PTMs marking early gametocytes (50). The histone prevalence increased significantly from early asexual development to schizonts in accord with higher histone gene expression, transcriptional activity, and DNA synthesis during schizogony (e.g., references 52, 53). The same study (50) discovered the H3K18me1 methylation mark and assigned it to the late stages of intraerythrocytic development. Later investigations found H3K18me1 in the centromeric regions of chromosomes, in particular, associated with internal *var* gene clusters (54). Because H3K18me1 was enriched in coding sequences and did not correlate with known activating marks, a function of this mark in chromosome organization during schizogony rather than a role in transcriptional regulation was suggested (54).

We applied loss-of-function studies, using a *Pf*SET10-KO and a *glmS* ribozyme-based *Pf*SET10-HA-KD line. The complete loss of *Pf*SET10 resulted in reduced intraerythrocytic growth [(38) and this study], while the partial reduction of *Pf*SET10 levels had no detectable effect on parasitemia. In addition, neither gametocyte commitment nor maturation was affected by *Pf*SET10 deficiency. These results were confirmed by Wyss et al. (39), showing that a rapamycin-inducible DiCre-dependent conditional *Pf*SET10-KO was neither able to impair blood stage replication nor gametocytogenesis. The differences in the loss-of-function phenotype during intraerythrocytic growth between the different transgenic lines may be based on the *Pf*SET10 levels (e.g., complete loss versus reduction). Similar contradictory results were obtained during loss-of-function approaches of the related chromatin-modifying protein *Pf*MORC (see detailed discussion of *Pf*MORC below). While partial *Pf*MORC knockdown using the *glmS*-ribozyme system had no significant effect on parasite survival (55), transposon mutagenesis identified *Pf*MORC as likely essential (56), and a TetR-DOZI knockdown system resulted in reduced intraerythrocytic growth and death of the parasite (46). We thus conclude that *Pf*SET10 has a crucial function for blood stage survival in a dose-dependent manner.

To unveil the *Pf*SET10 interaction network, we performed TurboID analyses and evaluated the hits via STRING analyses according to known protein-protein interaction patterns. We highlight multiple interactions of *Pf*SET10 with proteins involved in gene regulation, transcriptional control, and RNA processing, as well as DNA replication and repair, confirming the proposed function of *Pf*SET10 in chromatin modulation. Prominent interactors include, in addition to polymerases and helicases, epigenetic regulators like SET and bromodomain proteins, histone acetyltransferases, and deacetylases, but also MCM proteins and chromatin remodeling proteins like *Pf*ISWI, *Pf*SWIP, and *Pf*SNF2L. In addition, *Pf*SET10 associates with 14 out of the 28 known ApiAP2 transcription factors (57).

Large-scale protein interaction networks in *Plasmodium* schizonts were previously reported, with more than 20,000 putative protein interactions, organized into 600 protein clusters (58). Among others, chromatin regulator networks were identified that included the interaction of *Pf*MORC and an EELM2 domain-containing protein (PF3D7_1141800) with *Pf*AP2-P and the histone deacetylase *Pf*HDAC1, which was recently shown to be relevant for intraerythrocytic development and the regulation of genes encoding host cell invasion factors (59). Hillier and colleagues (58) hypothesized that *Pf*MORC and EELM2 proteins form a scaffold connecting transcription factor complexes with epigenetic regulators to affect nucleosome reorganization and regulate gene expression. Noteworthy, *Pf*AP2-P has been shown to bind to promoters of genes controlling trophozoite development, host cell remodeling, and antigenic variation and to also target a genetic element in the *var* gene introns that is important for tethering members of this multigene family to the nuclear periphery as part of their epigenetic silencing (47, 60). *P. falciparum* blood stages lacking *Pf*AP2-P exhibited de-repression of most *var* genes (47), and similar effects, that is, the upregulation of transcripts encoding multigene family proteins, were observed by us for *Pf*SET10-KO parasites. Both proteins, *Pf*AP2-P and *Pf*SET10, further interact with each other as well as with *Pf*HDAC1 and PF3D7_1141800 (47; this study), suggesting common functions of the two proteins in repressing genes assigned to antigenic variation and pathogenicity. Worth mentioning in this context while many malarial ApiAP2 proteins act as transcription factors, some of them bind heterochromatin independent of DNA motif recognition, where they act as activators or repressors of heterochromatic gene expression (61).

One of our top hits of *Pf*SET10 interactors is *Pf*MORC. MORC proteins belong to a highly conserved nuclear protein superfamily that is involved in signaling-dependent chromatin remodeling and epigenetic regulation (reviewed in references 62, 63). In plants, MORC proteins function in gene repression and heterochromatin compaction (64, 65). They can also form protein complexes with immune-responsive proteins, SWI chromatin remodeling proteins, and histone deacetylases (66–70). The *Toxoplasma gondii* ortholog *Tg*MORC was identified in a complex with *Tg*HDAC3 (the *Pf*HDAC1 homolog) and ApiAP2 proteins to support the epigenetic rewiring of sexual gene transcription (71–74). In *P. falciparum*, *Pf*MORC has been shown to associate with several ApiAP2 proteins, with which it shares DNA-binding sites (45, 48, 55, 58). In addition, *Pf*MORC locates together with *Pf*ISWI at *var* gene promoter regions, suggesting a role of the two proteins in *var* gene silencing (45). Recently, two independent interactomics analyses on *Pf*MORC have been published (46, 48). Among the reported interaction partners of *Pf*MORC were ApiAP2 transcription factors, EELM2 proteins, the RNA helicase *Pf*DBP5, the epigenetic regulators *Pf*BDP3, *Pf*HDAC1, *Pf*SET3, and *Pf*PHD2, as well as *Pf*ISWI (46, 48), all of which were also identified by us as *Pf*SET10 interactors.

Chromatin immunoprecipitation followed by deep sequencing has revealed that *Pf*MORC localizes to subtelomeric regions of all chromosomes across the *P. falciparum* genome, with additional occupancy at internal heterochromatic islands (46, 48). Within the subtelomeric regions of chromosomes, *Pf*MORC was bound upstream and within the gene bodies of multigene families, including *var, rif,* and *stevor* genes as well as genes encoding exported proteins. The majority of reads mapped to antigenic genes in the trophozoite and schizont stages. In addition, *Pf*MORC colocalized with the epigenetic mark H3K36me2, which is demarcated by the SET2 methyltransferase (26); a potential colocalization with H3K18me1 has not been investigated, though (48).

Phenotypical analyses by two independent loss-of-function approaches unveiled a significant upregulation of genes upon *Pf*MORC deficiency that are related to RBC invasion and host cell remodeling as well as to *var* genes (46, 48), and these observations are comparable to our reports on the transcriptional changes in the *Pf*SET10-KO line. Importantly, parasites lacking *Pf*MORC failed to maintain their overall chromatin structure with a significant weakening of the tightly controlled heterochromatin cluster (46).

We also performed a meta-analysis of the interactors of *Pf*SET10 and three of its interactors, that is, *Pf*MORC, *Pf*AP2-P, and *Pf*ISWI. We demonstrated an extensive network with 309 shared binding partners, forming clusters particularly assigned to DNA replication and transcription including RNA processing via the spliceosome. Furthermore, proteins involved in transcription and proteostasis, like proteasomal proteins and T-complex chaperonin subunits were found. A central role within this overarching chromatin modulation network plays *Pf*HDAC1, an interactor shared by all four bait proteins. In *T. gondii*, the *Pf*HDAC1 homolog *Tg*HDAC3 was shown to be recruited to *Tg*MORC in complex with various ApiAP2 transcription factors, resulting in chromatin remodeling and opening the access of genes for transcription, hence facilitating sexual differentiation (71, 72). Noteworthy, a weakness of the STRING analysis performed in this study is the missing information on the interactions between the chromatin-associated proteins identified here, like *Pf*MORC*, Pf*AP2-P*,* EELM2, *Pf*CHD1, *Pf*BDP3, *Pf*HDAC1, *Pf*SET3, *Pf*SET10, *Pf*PHD2, and *Pf*ISWI, which were therefore not correctly mapped.

Our data provide a first glimpse of the impact of *Pf*SET10 on regulating nucleic acid metabolism during intraerythrocytic replication and unveiled an extensive chromatin modulation network in the blood stage nuclei. The combined data on *Pf*SET10 and its top interacting proteins led us to propose two related critical functions of this network, that is, chromatin modulation and gene repression during intraerythrocytic development. Noteworthy, SET proteins present promising drug targets and HMT inhibitors are currently in clinical trials to test their use in cancer therapy. The most advanced clinical trials include tazemetostat, an Enhancer of zeste homolog 2 (EZH2) inhibitor approved for follicular lymphoma (75, 76). In *Plasmodium*, HMT inhibitors exhibit rapid antimalarial activities *in vitro* and *in vivo* (e.g., references 36, 77–80) and even a reversion of epigenetically acquired drug resistance by the HMT inhibitor chaetocin has been reported (81). Hence, information on proteins crucial during histone methylation in the *Plasmodium* blood and sexual stages may advance the current search for antiepigenetic targets in malaria therapy.

## MATERIALS AND METHODS

### Gene identifiers

The following PlasmoDB gene identifiers (gene IDs) are assigned to the genes and proteins investigated in this study: *Pf*SET10 (PF3D7_1221000); *Pf*39 (PF3D7_1108600); *Pf*92 (PF3D7_1364100); *Pf*s230 (PF3D7_0209000); STEVOR proteins (PF3D7_0300400; PF3D7_0324600; PF3D7_0631900; PF3D7_1254100); and histone H3 (PF3D7_0610400).

### Antibodies

The following antibodies were used: rabbit polyclonal anti-*Pf*39 antisera (82); rabbit polyclonal anti-*Pf*92 antisera (41); mouse and rabbit polyclonal anti-*Pf*s230 antisera (83); rabbit polyclonal anti-STEVOR antisera (84); mouse monoclonal anti-GFP antibody (Roche; Basel, CH); rat monoclonal anti-HA antibody (Roche); rabbit polyclonal antibodies directed against anti-H3K4me1, anti-H3K4me2, anti-H3K4me3, H3K18me1, anti-H3K36me1, anti-H3K36me2, anti-H3K36me3 antibody, and anti-H3 (Abclonal, Dusseldorf, Germany).

The following dilutions were used for (i) immunolabeling: mouse or rabbit anti-*Pf*s230 (1:200), rabbit anti-*Pf*92 (1:200), mouse anti-GFP (1:200), rat anti-HA (1:50), rabbit anti-H3K18me1 (1:500); (ii) immunoblotting: rabbit anti-*Pf*39 (1:10,000), mouse anti-GFP (1:200), rat anti-HA (1:50), rabbit anti-H3K4me3 (1:4,000), rabbit anti-H3K18me1 (1:4,000), rabbit anti-H3K4me1 (1:4,000), rabbit anti-H3K4me2 (1:4,000), rabbit anti-H3K4me3 (1:4,000), rabbit anti-H3K36me1 (1:4,000), rabbit anti-H3K36me2 (1:4,000), rabbit anti-H3K36me3 (1:4,000), rabbit anti-H3 (1:4,000), rabbit anti-STEVOR (1:2,000).

### Bioinformatics

Predictions of gene expression and protein properties and functions were made using the database PlasmoDB (http://plasmoDB.org) (85); the peak transcript expression of candidate genes was analyzed using table “Transcriptomes of 7 sexual and asexual life stages,” (11) and sex specificity was predicted using table “Gametocyte transcriptomes” (9) of the PlasmoDB database. The gene ontology enrichment (GO) analysis was performed using the ShinyGO 0.77 (86) with a *P*-value cutoff of 0.05. The network analyses were conducted using the STRING database (version 11.0) (87) and a Markov clustering algorithm with a confidence level of 0.9. For the meta-analysis of shared interactors, hits as provided by Table EV11 (45), Table S3a (46), Table S6 (47), and Table S2 (48) were compared with the *Pf*SET10 interactors (Table S6).

### Parasite culture

The gametocyte-producing strain *P. falciparum* NF54 (WT NF54) was cultured *in vitro* in RPMI1640/HEPES medium (Gibco; Thermo Fisher Scientific, Waltham, MA) supplemented with 10% (vol/vol) heat-inactivated human A+ serum, 50 µg/mL hypoxanthine (Sigma-Aldrich, Taufkirchen, Germany) and 10 µg/mL gentamicin (Gibco). For cultivation of the transgenic lines, the selection drug WR99210 (Jacobus Pharmaceutical Company, Princeton, NJ) was added at a final concentration of 4.0 nM. All cultures were kept at 37°C in an atmosphere of 5% O_2_ and 5% CO_2_ in N_2_. Human serum and erythrocyte concentrate were obtained from the Department of Transfusion Medicine, University Hospital Aachen, Germany. Donor sera and blood samples were pooled and anonymized. To synchronize the asexual blood stages, parasite cultures with 4% ring stages were centrifuged, and the pellet was resuspended in 5× pellet’s volume of 5% (wt/vol) sorbitol (AppliChem, Darmstadt, Germany)/ddH2O and incubated for 10 min at room temperature (RT). Cells were washed once with RPMI and cultivated as described. Schizonts and gametocytes were enriched via Percoll gradient centrifugation (GE Healthcare Life Sciences, Chicago, IL, USA) as previously described (88, 89). Gametocytogenesis was then induced by the addition of lysed RBCs (0.5 mL of 50% hematocrit lysed RBC in 15 mL of culture medium) followed by washing with RPMI the next day. To kill the asexual blood stages in gametocyte cultures, these were maintained in a cell culture medium supplemented with 50 mM N-acetylglucosamine (GlcNAc; Carl Roth, Karlsruhe, Germany) for five consecutive days (90).

### Generation of the *Pf*SET10-HA-KD parasite line

The *Pf*SET10-HA-KD line was generated by single-crossover homologous recombination, using the pSLI-HA-*glmS* vector (41). A 906-bp gene fragment homologous to the 3′-coding region of the *pfset10* gene was amplified using forward primer *Pf*SET10 pSLI-HA-*glmS* SacII and reverse primer *Pf*SET10 pSLI-HA-*glmS* XhoI. The stop codon was excluded from the homologous gene fragment. The insert was ligated to the vector backbone via SacII and XhoI restriction sites. A WT NF54 culture with at least 5% ring stages was transfected with 100 µg plasmid DNA in transfection buffer by electroporation (310 V, 950 µF, 12 ms; Bio-Rad gene-pulser Xcell) as described (e.g., references 91, 92). At 6 hours post-transfection, WR99210 was introduced at a final concentration of 4 nM. After 3–5 weeks, WR99210-resistant parasites emerged in the cultures. WT NF54 parasites were eliminated from the culture by the addition of neomycin (G418; Sigma-Aldrich) at a final concentration of 550 µg/mL for a maximum of 7 days. Successful integration into the gene locus was confirmed by diagnostic PCR using primers 5′ Int *Pf*SET10 pSLI-HA-*glmS* (i), 3′ Int *Pf*SET10 pSLI-HA-*glmS* (ii), pSLI-HA-*glmS* FP (iii), and pSLI-HA-*glmS* RP (iv) (Fig. S1A and B; for primer sequences, see Table S7).

### Generation of the *Pf*SET10-TurboID-GFP line

The *Pf*SET10-TurboID-GFP line was generated by single-crossover homologous recombination, using the pSLI-TurboID-GFP vector (44). A 1,144-bp gene fragment homologous to the 3′-coding region of the *pfset10* gene was amplified using forward primer *Pf*SET10 pSLI-TurboID-GFP NotI FP and reverse primer *Pf*SET10 pSLI-TurboID-GFP SpeI RP. The stop codon was excluded from the full-length gene sequence. The fragment was ligated to the vector via NotI and SpeI restriction sites. Transfection and selection were carried out as described above. Successful integration into the gene locus was confirmed by diagnostic PCR using primers 5′ Int *Pf*SET10 pSLI-TurboID-GFP (i), 3′ Int *Pf*SET10 pSLI-TurboID-GFP (ii), pSLI-HA- *glmS* FP (iii), and pSLI-TurboID-GFP (iv) (Fig. S6A and B; for primer sequences, see Table S7).

### Indirect immunofluorescence assay

Parasite cultures containing mixed asexual blood stages and gametocytes of WT NF54 as well as of lines *Pf*SET10-HA-KD and *Pf*SET10-TurboID-GFP were coated on glass slides as cell monolayers and then air-dried. After fixation in methanol at −80°C for 10 min, the cells were serially incubated in 0.01% (wt/vol) saponin/0.5% (wt/vol) BSA/PBS, and 1% (vol/vol) neutral goat serum (Sigma-Aldrich)/PBS for 30 min at RT to facilitate membrane permeabilization and blocking of non-specific binding. The primary antibodies were diluted in 3% (wt/vol) BSA/PBS and were added to the slide for 2-h incubation at 37°C. The slides were washed 3× with PBS and incubated with the secondary antibody for 1 h at 37°C. Following 2× washing with PBS, the incubation with the second primary antibody and the corresponding visualization with the second secondary antibody were carried out as described above. The nuclei were stained with Hoechst 33342 staining solution for 2 min at RT (1:5,000 in 1× PBS). The cells were mounted with an anti-fading solution (Citifluor Limited, London, UK), covered with a coverslip, and sealed airtight with nail polish. The parasites were visualized by conventional fluorescence microscopy using a Leica DM5500 B (Leica, Wetzlar, Germany) microscope. The following secondary antibodies were used: anti-mouse Alexa Fluor 488, anti-rabbit Alexa Fluor 488, anti-rat Alexa Fluor 488, anti-mouse Alexa Fluor 594, anti-rabbit Alexa Fluor 594 (1:1,000; all fluorophores from Invitrogen Molecular Probes, Eugene, OR, or Sigma-Aldrich); further Alexa Fluor 594 streptavidin (1:500; Invitrogen Molecular Probes) was used. Alternatively, the asexual blood stages were stained with 0.01% (wt/vol) Evans Blue (Sigma-Aldrich)/PBS for 3 min at RT followed by 5 min washing with PBS. Images were processed using the Adobe Photoshop CS software.

### Western blotting

Asexual blood stage parasites of WT NF54 as well as lines *Pf*SET10-KO, *Pf*SET10-HA-KD, and *Pf*SET10-TurboID-GFP were harvested from mixed or synchronized cultures, while gametocytes were enriched by Percoll purification. The infected RBCs were lysed with 0.05% (wt/vol) saponin/PBS for 10 min at 4°C to release the parasites, then washed with PBS, and resuspended in lysis buffer (0.5% [vol/vol] Triton X-100, 4% [wt/vol] SDS in PBS) supplemented with protease inhibitor cocktail. After adding 5× SDS-PAGE loading buffer containing 25 mM DTT, the lysates were heat-denatured for 10 min at 95°C. Lysates were separated via SDS-PAGE and transferred to Hybond ECL nitrocellulose membranes (Amersham Biosciences, Buckinhamshire, UK) following the manufacturer’s protocol. Non-specific binding sites were blocked by incubation with 5% (wt/vol) skim milk and 1% (wt/vol) BSA in Tris buffer (pH 7.5) for 1 h at 4°C. For immunodetection, membranes were incubated overnight at 4°C with the primary antibody in 3% (wt/vol) skim milk/TBS. The membranes were washed 3× each with 3% (wt/vol) skim milk/TBS and 3% (wt/vol) skim milk/0.1% (vol/vol) Tween/TBS and then incubated for 1 h at RT with goat anti-mouse, anti-rabbit, or anti-rat alkaline phosphatase-conjugated secondary antibodies (dilution 1:10,000, Sigma-Aldrich) in 3% (wt/vol) skim milk/TBS. The membranes were developed in a NBT/BCIP solution (nitroblue tetrazolium chloride/5-bromo-4-chloro-3-indoxyl phosphate; Roche) for up to 20 min at RT. For the detection of biotinylated proteins, the blocking step was performed overnight at 4°C in 5% (wt/vol) skim milk/TBS, and the membrane was washed 5× with 1× TBS before incubation with streptavidin-conjugated alkaline phosphatase (dilution 1:1,000, Sigma-Aldrich) in 5% (wt/vol) BSA/TBS for 1 h at RT. Blots were scanned and processed using the Adobe Photoshop CS software. Band intensities were measured using the ImageJ program version 1.51f.

### Asexual blood stage replication assay

#### *Pf*SET10-KO

The asexual blood stage replication assay was performed as described (38). Sorbitol-synchronized asexual blood stage cultures of line *Pf*SET10-KO and WT NF54 were set to an initial parasitemia of 0.25% at the ring stage and cultivated as described. Giemsa-stained thin blood smears were prepared every 12 hours over an 84-hour period. The parasitemia at each time point was determined microscopically at 1,000× magnification by counting the percentage of parasites in 1,000 RBCs. Blood stages (ring, trophozoites, and schizonts) present in the cultures were identified at each time point by counting 50 infected RBCs per set. Two experiments were performed, each in triplicate. Data analysis was performed using MS Excel 2016 and GraphPad Prism 5.

#### *Pf*SET10-HA-KD

Asexual blood stage cultures of line *Pf*SET10-HA-*glmS* and WT NF54 were Sorbitol-synchronized and set to an initial parasitemia of 0.25% ring stages. The cultures were then continuously treated with 2.5 mM GlcN (Sigma-Aldrich) for transcript knockdown. Untreated cultures were used for control. The parasitemia was assessed every 12 hours during a period of 96 hours employing the LUNA-FX7 automated cell counter (logos biosystems, Dongan-gu Anyang-si, Republic of Korea). Two experiments were performed, each in triplicate. Data analysis was performed using MS Excel 2016 and GraphPad Prism 5.

### Gametocyte commitment and development assays

#### *Pf*SET10-KO

The gametocyte development assay was performed as described (93). Sorbitol-synchronized cultures of line *Pf*SET10-KO and WT NF54 were set to a parasitemia of 5% ring stages. Gametocytogenesis was induced by the addition of lysed RBCs followed by washing with RPMI the next day. The cultures were maintained in cell culture medium supplemented with 50 mM GlcNAc to kill the asexual blood stages for 5 days and then maintained with normal cell culture medium until day 10 post-induction. Giemsa-stained blood smears were prepared every 48 hours between day 5 and day 13 post-induction. The gametocytemia at each time point was determined microscopically by counting the percentage of gametocytes in 1,000 RBCs. Two experiments were performed, each in triplicate. Data were analyzed using MS Excel 2016 and GraphPad Prism 5.

#### *Pf*SET10-HA-KD

The gametocyte commitment and development assay was performed as described (94). Sorbitol-synchronized cultures of line *Pf*SET10-HA-KD and WT NF54 were each divided into three groups (untreated, pre-induction, and post-induction group) and set to an initial parasitemia of 0.5% schizonts. The pre-induction group was cultured in a medium supplemented with 2.5 mM GlcN for 72 hours. Then, all cultures were adjusted to a parasitemia of 5.3% and gametocytogenesis was induced by the addition of lysed RBC for 24 hours. Afterward, cells were washed and the parasites were cultivated in a medium for 4 days. Heparin was added to the medium at a final concentration of 20 U/mL to kill the asexual blood stages. The post-induction group was treated with 2.5 mM GlcN for seven consecutive days after the addition of lysed RBC for 24 hours. Giemsa-stained thin blood smears were prepared on days 3 and 7 post-induction and gametocytemia was determined in 1,000 RBCs. Three experiments were performed, each in triplicate. Data were analyzed using MS Excel 2016 and GraphPad Prism 5.

### RNA isolation and sequencing

*Pf*SET10-KO and WT NF54 schizonts were enriched by Percoll gradient centrifugation (GE Healthcare Life Sciences, Chicago, IL, USA) as described above. Purified samples were stored at −80°C in TRIzol LS reagent (Life Technologies, Thermo Fisher Scientific) and RNA was isolated according to the manufacturer’s protocol. RNA preparations were treated with RNase-free DNase I (Qiagen, Hilden, Germany) to remove gDNA contamination, followed by phenol/chloroform extraction and ethanol precipitation. The quality of the RNA was assessed by ND-1000 (NanoDrop Technologies; Thermo Fisher Scientific) and agarose gel electrophoresis. RNA purity was confirmed by an A260/A280 ratio above 2.1. Purified RNA underwent mRNA sequencing on the Illumina NextSeq sequencer (Genomic Facility of the Interdisciplinary Center for Clinical Research, University Hospital Aachen, Germany) using standard paired-end sequencing protocols provided by Illumina. The data underwent analysis using the NextGen pipeline, an in-house adapted pipeline integrated into the workflow management system of the QuickNGS-Environment, as described (93). Significantly deregulated genes were defined as ≥2-fold deregulated in their transcript levels (upregulated genes: log2 fold change ≥ 1; downregulated genes: log2 fold change ≤ −1).

### Preparation of samples for TurboID analysis

Highly synchronized schizont cultures of line *Pf*SET10-TurboID-GFP and WT NF54 as control were treated with biotin at a final concentration of 50 µM for 10 min to induce the biotinylation of proximal proteins by the BirA ligase. After treatment, the RBCs were lysed with 0.05% (wt/vol) saponin, and the parasites were resuspended in 100 µL binding buffer (Tris buffer containing 1% [vol/vol] Triton X-100 and protease inhibitor). The sample was sonicated on ice (2 × 60 pulses at 30% duty cycle) and another 100 µL of ice-cold binding buffer was added. After a second session of sonification, cell debris was pelleted by centrifugation (5 min, 16,000 × *g*, 4°C). The supernatant was mixed with pre-equilibrated Cytiva Streptavidin Mag Sepharose Magnet-Beads (Cytiva, Washington, DC, USA) in a low-binding reaction tube. Incubation was performed with slow end-over-end mixing overnight at 4°C. The beads were washed 6× with 500 µL washing buffer (3× RIPA buffer containing 0.03% [wt/vol] SDS, followed by 3 × 25 mM Tris buffer [pH 7.5]) and were resuspended in 45 µL elution buffer (1% [wt/vol] SDS/5 mM biotin in Tris buffer [pH 7.5]), followed by an incubation for 5 min at 95°C. The supernatant was transferred into a new reaction tube and stored at −20°C. For each culture, three independent samples were collected.

### Proteolytic digestion

Samples were processed by single-pot solid-phase-enhanced sample preparation (SP3) as described (95, 96). Proteins bound to the streptavidin beads were released by incubating the samples for 5 min at 95°C in an SDS-containing buffer (1% [wt/vol] SDS, 5 mM biotin in water/Tris buffer, pH 8.0). After elution, proteins were reduced and alkylated, using DTT and iodoacetamide (IAA), respectively. Afterward, 2 µL of carboxylate-modified paramagnetic beads (Sera-Mag Speed Beads, GE Healthcare, Chicago, IL; 0.5 µg solids/μL in water as described [95]) was added to the samples. After adding acetonitrile to a final concentration of 70% (vol/vol), samples were allowed to settle at RT for 20 min. Subsequently, beads were washed twice with 70% (vol/vol) ethanol in water and once with acetonitrile. The beads were resuspended in 50 mM NH_4_HCO_3_ supplemented with trypsin (Mass Spectrometry Grade, Promega, Madison, WI) at an enzyme-to-protein ratio of 1:25 (wt/wt) and incubated overnight at 37°C. After overnight digestion, acetonitrile was added to the samples to reach a final concentration of 95% (vol/vol) followed by incubation at RT for 20 min. To increase the yield, supernatants derived from this initial peptide-binding step were additionally subjected to the SP3 peptide purification procedure (96). Each sample was washed with acetonitrile. To recover bound peptides, paramagnetic beads from the original sample and corresponding supernatants were pooled in 2% (vol/vol) dimethyl sulfoxide (DMSO) in water and sonicated for 1 min. After 2 min of centrifugation at 14,000 × *g* and 4°C, supernatants containing tryptic peptides were transferred into a glass vial for MS analysis and acidified with 0.1% (vol/vol) formic acid.

### Liquid chromatography-mass spectrometry analysis

Tryptic peptides were separated using an Ultimate 3000 RSLCnano LC system (Thermo Fisher Scientific) equipped with a PEPMAP100 C18 5 µm 0.3 × 5 mm trap (Thermo Fisher Scientific) and an HSS-T3 C18 1.8 µm, 75 µm × 250 mm analytical reversed-phase column (Waters Corporation, Milford, MA). Mobile phase A was water containing 0.1% (vol/vol) formic acid and 3% (vol/vol) DMSO. Peptides were separated by running a gradient of 2%–35% mobile phase B (0.1% [vol/vol] formic acid, 3% [vol/vol] DMSO in ACN) over 40 min at a flow rate of 300 nL/min. Total analysis time was 60 min including wash and column re-equilibration steps. Column temperature was set to 55°C. Mass spectrometric analysis of eluting peptides was conducted on an Orbitrap Exploris 480 (Thermo Fisher Scientific) instrument platform. The spray voltage was set to 1.8 kV, the funnel RF level to 40, and the heated capillary temperature was at 275°C. Data were acquired in data-dependent acquisition (DDA) mode targeting the 10 most abundant peptides for fragmentation (Top10). Full MS resolution was set to 120,000 at *m/z* 200 and full MS automated gain control (AGC) target to 300% with a maximum injection time of 50 ms. The mass range was set to *m/z* 350–1,500. For MS2 scans, the collection of isolated peptide precursors was limited by an ion target of 1 × 10^5^ (AGC target value of 100%) and maximum injection times of 25 ms. Fragment ion spectra were acquired at a resolution of 15,000 at *m/z* 200. The intensity threshold was kept at 1E4. The isolation window width of the quadrupole was set to 1.6 *m/z* and the normalized collision energy was fixed at 30%. All data were acquired in profile mode using positive polarity. Each sample was analyzed in three technical replicates.

### Data analysis and label-free quantification

DDA raw data acquired with the Exploris 480 were processed with MaxQuant (version 2.0.1) (97, 98), using the standard settings and label-free quantification (LFQ) enabled for each parameter group, that is, control and affinity-purified samples (LFQ min ratio count 2, stabilize large LFQ ratios disabled, match-between-runs). Data were searched against the forward and reverse sequences of the *P. falciparum* proteome (UniProtKB/TrEMBL, 5,445 entries, UP000001450, released April 2020) and a list of common contaminants. For peptide identification, trypsin was set as protease allowing two missed cleavages. Carbamidomethylation was set as fixed and oxidation of methionine as well as acetylation of protein N-termini as variable modifications. Only peptides with a minimum length of 7 amino acids were considered. Peptide and protein false discovery rates (FDR) were set to 1%. In addition, proteins had to be identified by at least two peptides. Statistical analysis of the data was conducted using Student’s *t*-test, which was corrected by the Benjamini-Hochberg (BH) method for multiple hypothesis testing (FDR of 0.01). In addition, proteins in the affinity-enriched samples had to be identified in all three biological replicates and show at least a twofold enrichment compared to the controls. The data sets of protein hits were further edited by verification of the gene IDs and gene names via the PlasmoDB database. PlasmoDB gene IDs were extracted from the fasta headers provided by mass spectrometry and verified manually. Following the generation of an initial list of significantly enriched proteins, those with a putative signal peptide were excluded. For STRING-based analyses, only proteins with a log2 ratio ≥ 23 were considered. A Markov clustering algorithm with a confidence level of 0.9 was applied.

### Statistical analysis

Data are expressed as mean ± SD. Statistical differences were determined using Student’s *t*-test type 1. *P*-values ≤ 0.05 were considered statistically significant and are represented in the figures as *P ≤ 0.05; ***P ≤ 0.001.

## Data Availability

The mass spectrometry proteomics data have been deposited to the ProteomeXchange Consortium (http://proteomecentral.proteomexchange.org) via the jPOST partner repository (99) with the data set identifiers PXD052931 (ProteomeXchange) and JPST003166 (jPOST).

## SUPPLEMENTAL MATERIAL

Supplemental Figures msphere.00495-24-s0001.pdf Figures S1 to S9.

Table S1 msphere.00495-24-s0002.xlsx Comparative transcriptomics.

Table S2 msphere.00495-24-s0003.xlsx TurboID peptide counts.

Table S3 msphere.00495-24-s0004.xlsx TurboID hits.

Table S4 msphere.00495-24-s0005.xlsx Select PfSET10 interactors grouped by function.

Table S5 msphere.00495-24-s0006.xlsx TurboID string PfSET10 interactors.

Table S6 msphere.00495-24-s0007.xlsx String chromatin modulator interaction network.

Table S7 msphere.00495-24-s0008.pdf Primer list.

ASM does not own the copyrights to Supplemental Material that may be linked to, or accessed through, an article. The authors have granted ASM a non-exclusive, world-wide license to publish the Supplemental Material files. Please contact the corresponding author directly for reuse.
