# Supplementary material for: The Plasmodium falciparum histone methyltransferase SET10 participates in a chromatin modulation network crucial for intraerythrocytic development
Source: mSphere. 2024 Oct 24;9(11):e00495-24. doi: 10.1128/msphere.00495-24 (PMC11580448; doi:10.1128/msphere.00495-24)
Supplement: Supplemental Figures — Figures S1 to S9. [file msphere.00495-24-s0001.pdf]

The *Plasmodium falciparum* histone methyltransferase SET10 participates in a chromatin modulation network crucial for intraerythrocytic development

Jean-Pierre Musabyimana, Sherihan Musa, Janice Manti, Ute Distler, Stefan Tenzer, Che Julius Ngwa, and Gabriele Pradel

Supplemental Figures

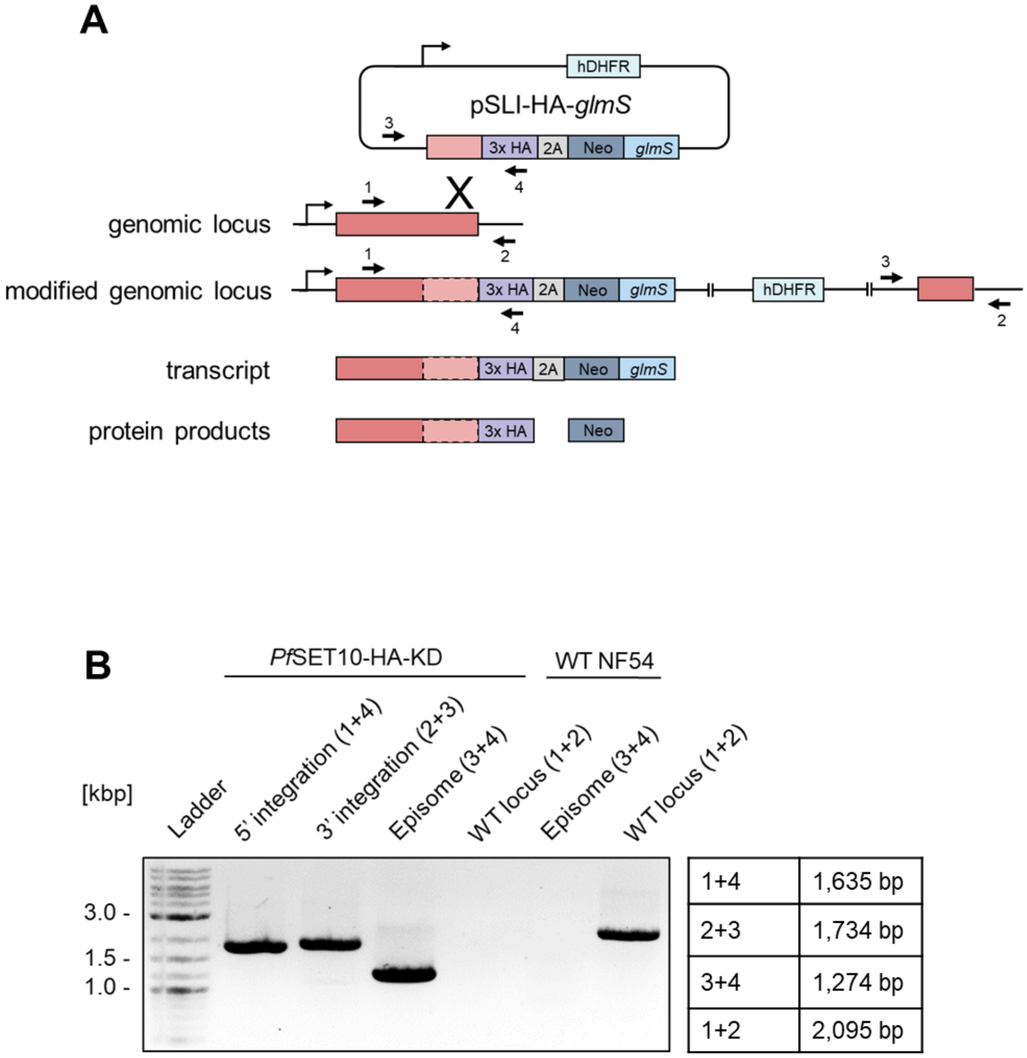

Figure S1. Generation of the *Pf*SET10-HA-KD line. (A) Schematic depicting the single-crossover homologous recombination strategy for the generation of the pSLI-HA-*glmS*-based *Pf*SET10-HA-KD line. The coding region of the gene of interest was fused at the 3'-end with a HA-encoding sequence followed by the 2A-skip peptide sequence and the Neo and *glmS*-ribozyme sequences. The numbered arrows indicate the positions of primers used to confirm vector integration. *glmS*, glucosamine-6-phosphate-activated ribozyme; HA, hemagglutinin; hDHFR, human dihydrofolate reductase-encoding gene conferring resistance to WR99210; Neo, gene conferring resistance to neomycin. **(B)** Confirmation of vector integration into the *pfset10* gene locus. Diagnostic PCR demonstrates successful 5′ (primers 1 and 4) and 3′ (primers 3 and 2) integration in line *Pf*SET10-HA-KD. WT NF54 gDNA served as control, demonstrating the original gene locus (primers 1 and 2). Episomal DNA was further detected (primers 3 and 4). Band sizes are indicated.

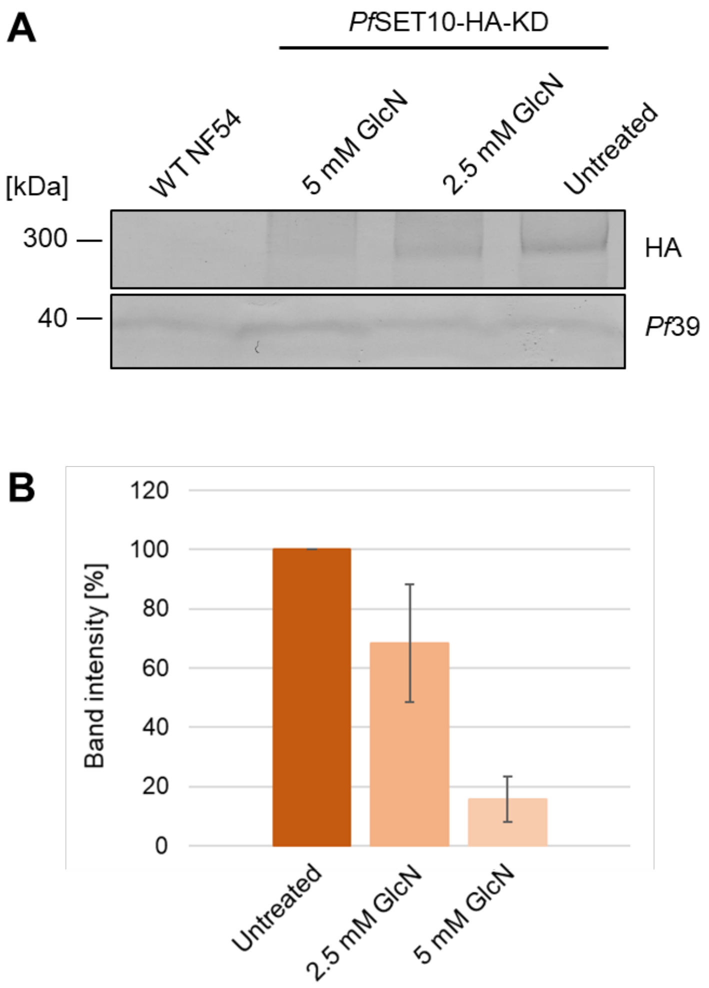

Figure S2. Verification of *Pf*SET10-HA knockdown. (A) Confirmation of *Pf*SET10-HA knockdown. Lysates of mixed asexual blood stages of line *Pf*SET10-HA-KD treated with 2.5 and 5 mM GlcN for 72 h were subjected to Western blotting using rat anti-HA antibody to detect *Pf*SET10-HA (~275 kDa). Untreated *Pf*SET10-HA-KD parasites and WT NF54 served as controls. Equal loading was confirmed by immunoblotting with rabbit antisera against *Pf*39 (~39 kDa). **(B)** Quantification of *Pf*SET10-HA levels following knockdown. *Pf*SET10-HA band intensities of three independent Western blots as described in (A) were quantified using Image J and normalized to the respective *Pf*39 protein band intensities (untreated set to 100%). Results are shown as mean ± SD.

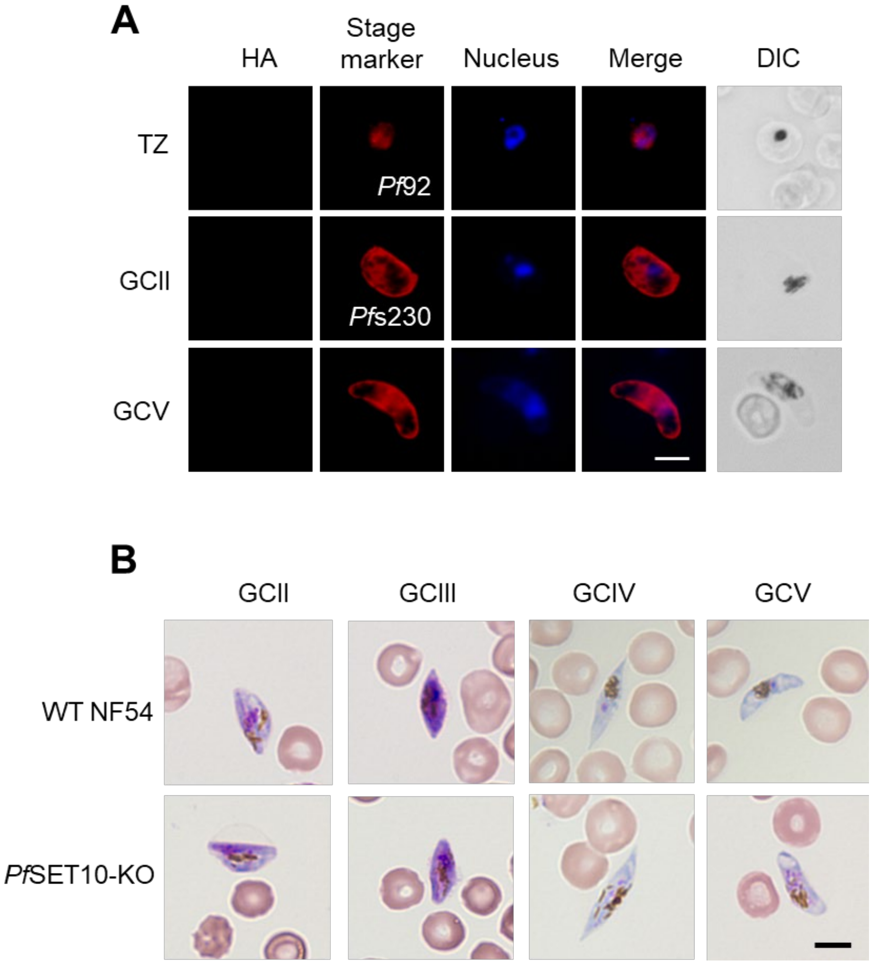

Figure S3. *Pf*SET10-HA immunolabelling control and *Pf*SET10-KO gametocyte morphology. (**A**) Immunofluorescence assay control for line *Pf*SET10-HA-KD. Methanol-fixed WT NF54 trophozoites (TZ) and gametocyte (GC) stages II and V were immunolabeled with rat anti-HA antibody (green). Asexual blood stages and gametocytes were highlighted using rabbit antisera directed against *Pf*92 and *Pf*s230, respectively (red); nuclei were highlighted with Hoechst 33342 nuclear stain (blue). DIC, differential interference contrast. Bar, 5 µm. **(B)** Morphology of *Pf*SET10-KO gametocytes. Gametocyte (GC) stages II-V of line *Pf*SET10-KO and WT NF54 were Giemsa-stained to compare their morphologies. Bar, 5 µm.

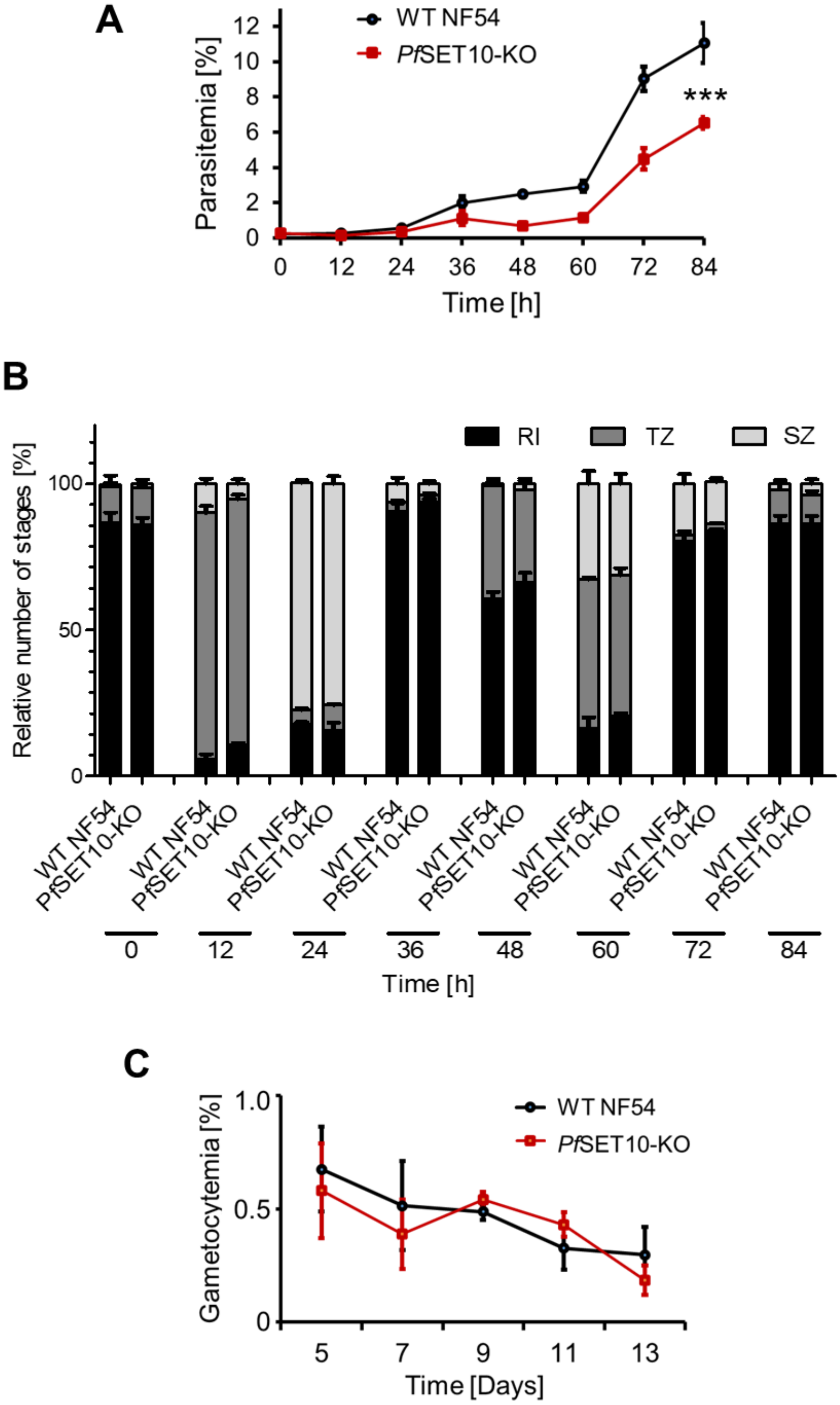

Figure S4. Intraerythrocytic growth and gametocyte development of the *Pf*SET10-KO line. (**A**) Intraerythrocytic growth of line *Pf*SET10-KO. Synchronized ring stage cultures of line *Pf*SET10-KO and WT NF54 were set up with an initial parasitemia of 0.25%. Parasitemia was followed via Giemsa smears over a time-period of 0 - 84 h. The experiment was performed in triplicate (mean ± SD). Significant differences in parasitemia between *Pf*SET10-KO and WT NF54 are indicated (***$p \leq 0.001$; Student's t-test). (**B**) Stage development of the *Pf*SET10-KO blood stages. Parasites were set up as described in (A) and the numbers of rings (RI),

trophozoites (TZ), and schizonts (SZ) were determined in a total number of 50 infected RBCs every 24 h via Giemsa-stained blood smears. The experiment was performed in triplicate (mean ± SD). (**C**) Gametocyte development of line *Pf*SET10-KO. Gametocyte production was induced in synchronized ring stage cultures of line *Pf*SET10-KO and WT NF54 at a parasitemia of 5.3% by addition of lysed RBCs and the gametocytemia was followed via Giemsa smears every 48 h between day 5 - 13 post-induction. The experiment was performed in triplicate (mean ± SD).

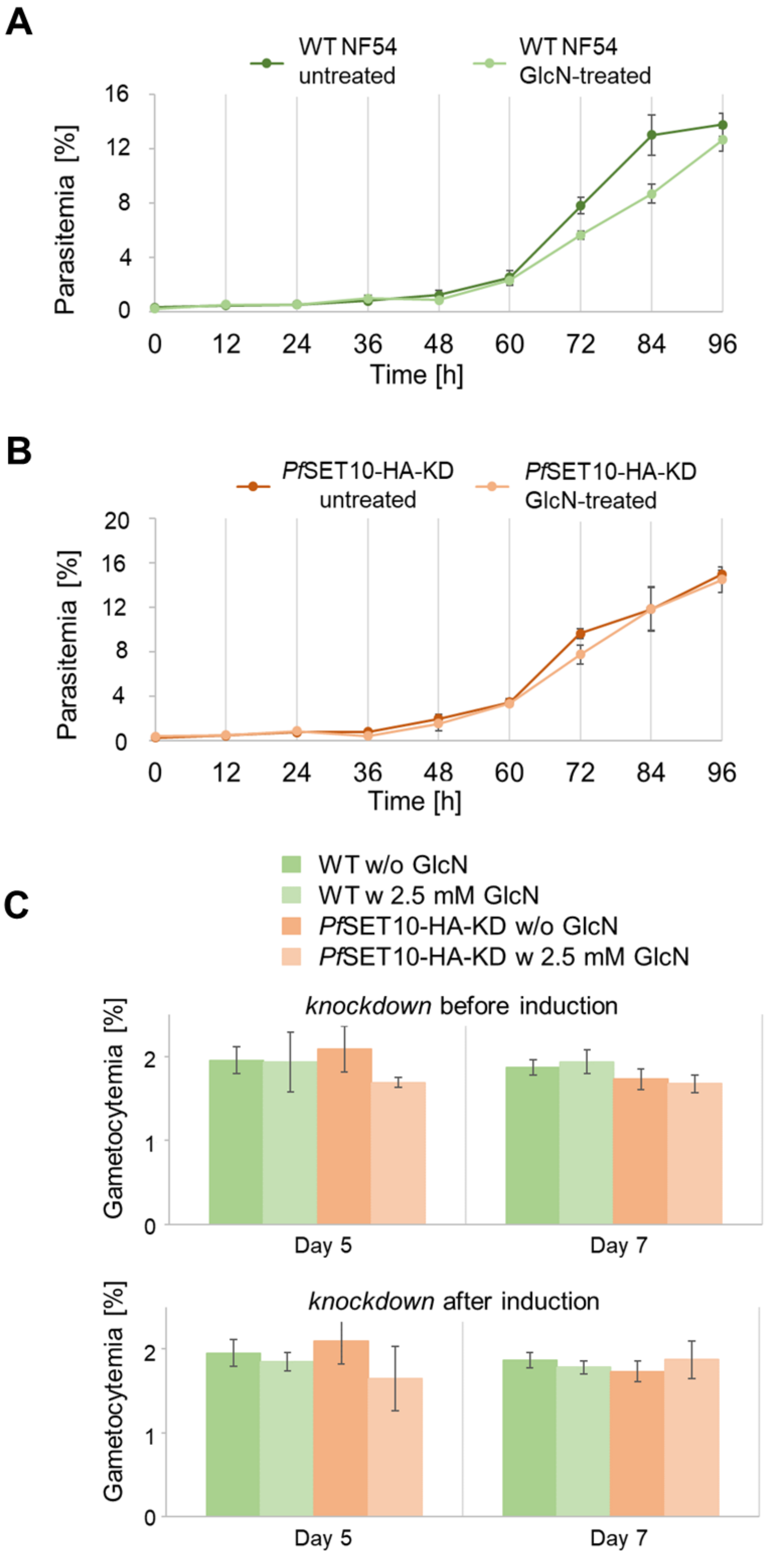

Figure S5. Intraerythrocytic growth and gametocyte formation following *Pf*SET10-HA knockdown. (**A**, **B**) Intraerythrocytic growth following *Pf*SET10-HA knockdown. Synchronized ring stage cultures of line *Pf*SET10-HA-KD (**A**) and WT NF54 (**B**) with a starting parasitemia of 0.25% were maintained in cell culture medium supplemented or not with 2.5 mM GlcN for transcript knockdown. The parasitemia was followed via Giemsa smears over a time-period of 0 - 96 h. The experiment was performed in triplicate (mean ± SD). (**C**)

Gametocyte formation in dependence of *Pf*SET10-HA knockdown. Synchronized parasites of line *Pf*SET10-HA-KD and WT NF54 were cultivated in the presence (knockdown before induction) or absence (knockdown after induction) of 2.5 mM GlcN. Gametocyte production was then induced by addition of lysed RBCs. The cultures were maintained in cell culture medium supplemented or not with 2.5 mM GlcN and the gametocytemia was determined via Giemsa smears on days 5 and 7 post-induction. The experiment was performed in triplicate (mean ± SD).

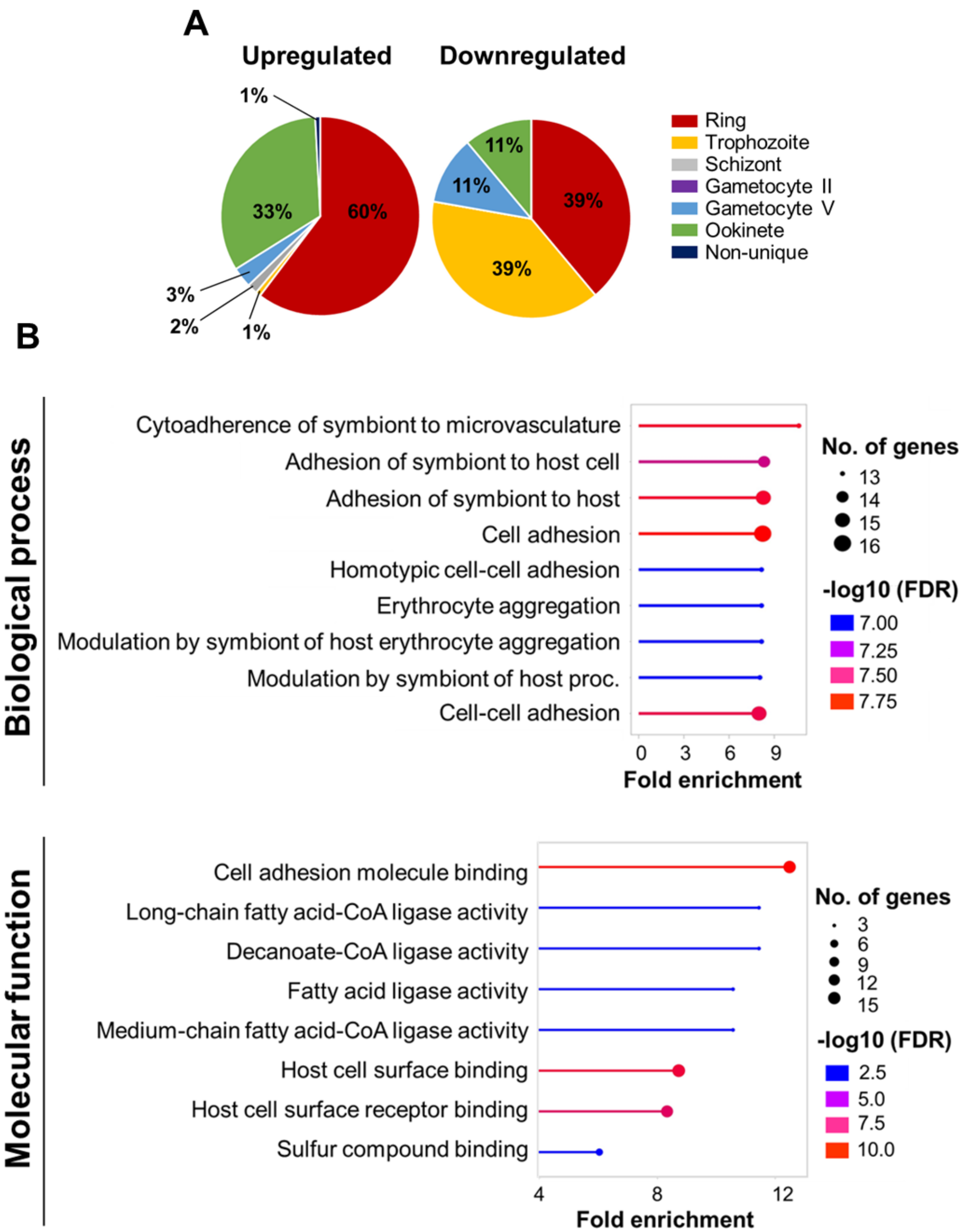

Figure S6. Deregulation of genes in the *Pf*SET10-KO line. (**A**) Pie chart depicting percentages of deregulated genes in line *Pf*SET10-KO grouped by peak transcript expression in seven asexual and sexual lifecycle stages (PlasmoDB). (**B**) Functional prediction analysis of upregulated genes in the *Pf*SET10-KO. A GO enrichment analysis of the transcriptionally upregulated genes was performed using ShinyGO program ($p < 0.05$) and the enriched GO terms were based on biological process and molecular function.

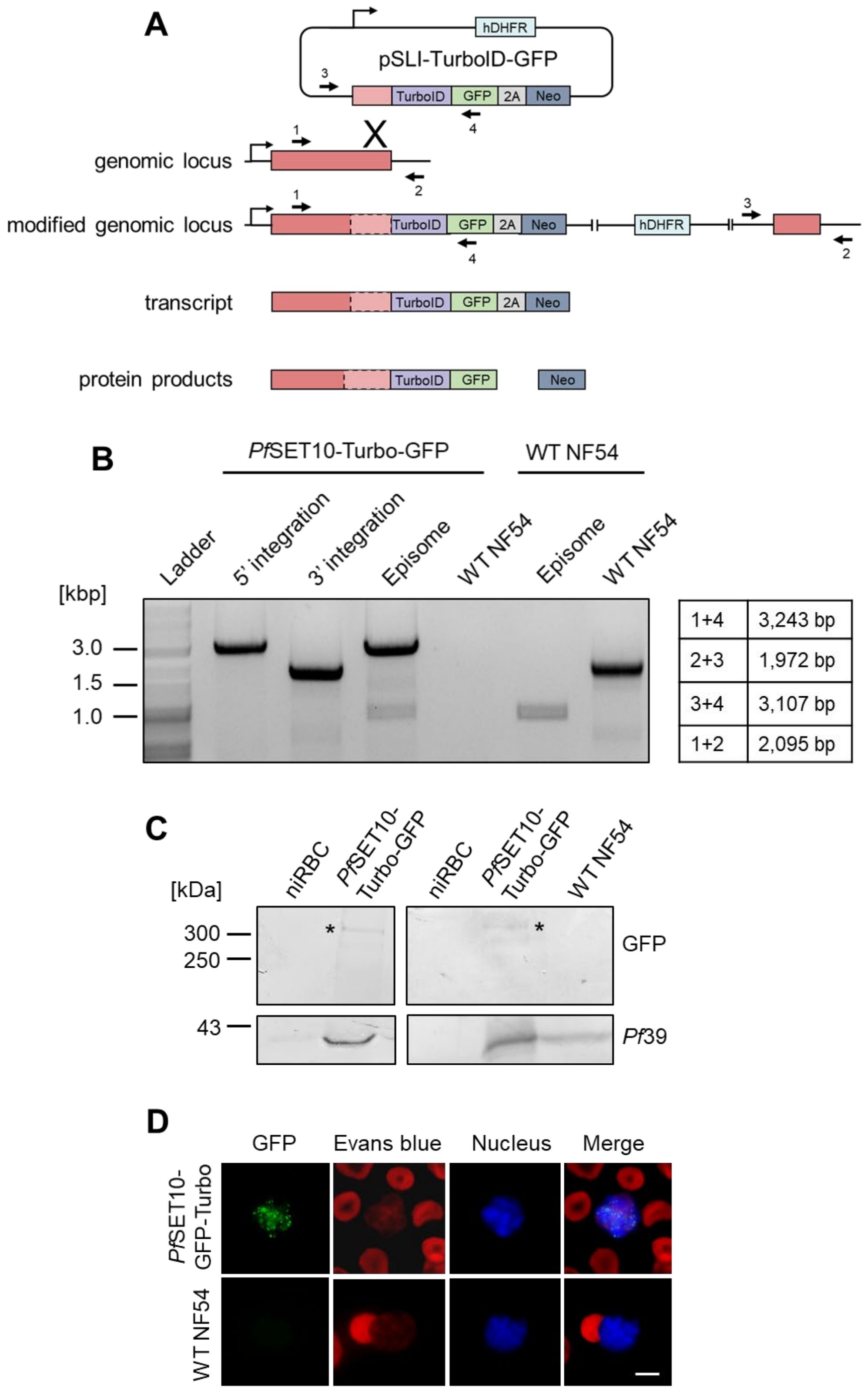

Figure S7. Generation of the *Pf*SET10-TurboID-GFP line. (**A**) Schematic depicting the single-crossover homologous recombination strategy for the generation of the pSLI-TurboID-GFP-based line. The coding region of the gene of interest was fused at the 3'-end with a sequence coding for an advanced *E. coli* biotin ligase and a GFP-encoding sequence followed by the 2A-skip peptide sequence and the Neo sequence. The numbered arrows indicate the

positions of primers used to confirm vector integration. HA, hemagglutinin; hDHFR, human dihydrofolate reductase-encoding gene conferring resistance to WR99210; Neo, gene conferring resistance to neomycin. **(B)** Confirmation of vector integration into the *pfset10* gene locus. Diagnostic PCR demonstrates successful 5′ (primers 1 and 4) and 3′ (primers 3 and 2) integration. As a control, WT NF54 gDNA was used, demonstrating the original gene locus (primers 1 and 2). Episomal DNA was further detected (primers 3 and 4). Band sizes are indicated. (**C**) Expression of *Pf*SET10-TurboID-GFP. Asexual blood stage lysates of line *Pf*SET10-TurboID-GFP were immunoblotted with mouse anti-GFP antibody to detect *Pf*SET10-TurboID-GFP (~300 kDa). Non-infected RBCs (niRBC) and WT NF54 served as negative controls; equal loading was confirmed by immunoblotting with rabbit antisera against *Pf*39 (~39 kDa). Asterisk highlights the *Pf*SET10-TurboID-GFP protein. (**D**) Localization of *Pf*SET10-TurboID-GFP in transgenic schizonts. Methanol-fixed schizonts of line *Pf*SET10-TurboID-GFP and WT NF54 were immunolabeled with anti-GFP antibody (green); schizonts were highlighted using Evans Blue (red) and nuclei were highlighted with Hoechst 33342 nuclear stain (blue). Bar, 5 µm.

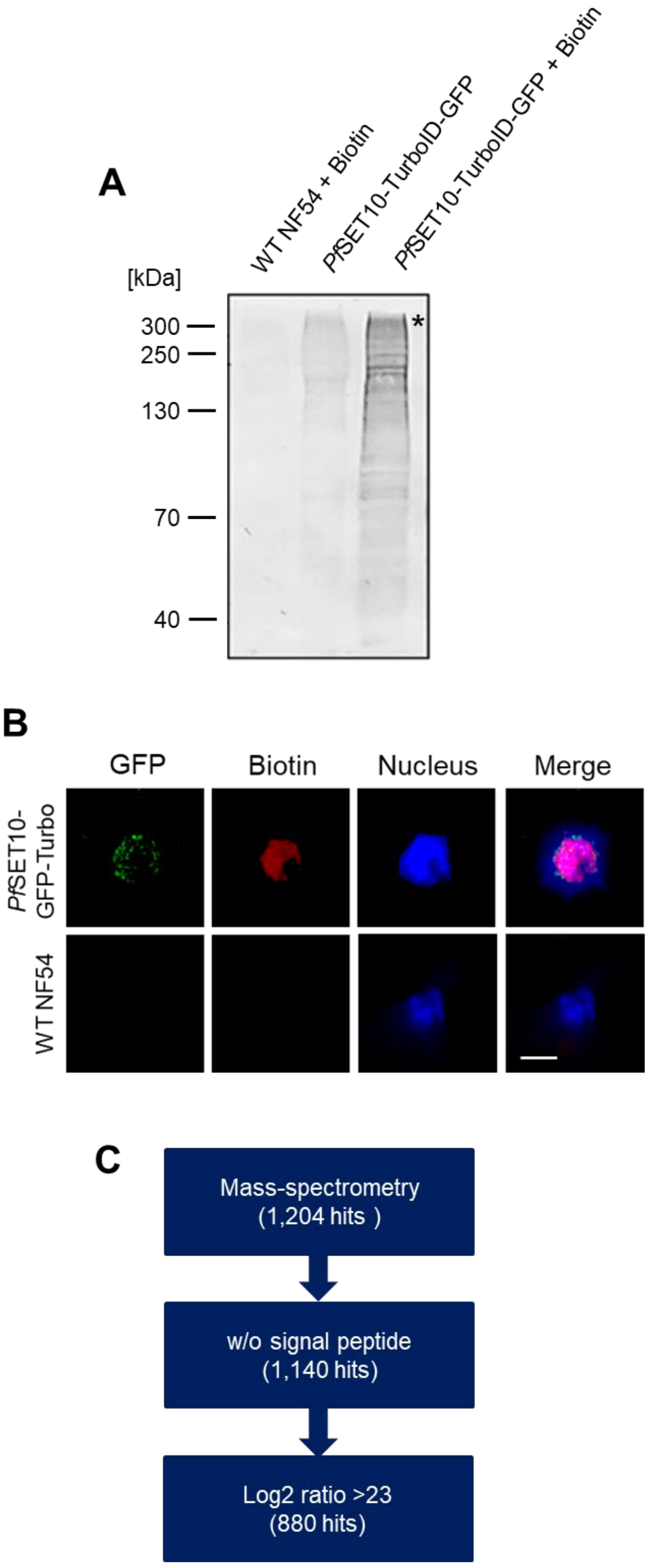

Figure S8. Verification of biotinylated proteins in the *Pf*SET10-TurboID-GFP line. (**A**) Detection of biotinylated proteins. Asexual blood stages of line *Pf*SET10-TurboID-GFP were treated or not with 50 µM biotin for 10 min. Lysates were prepared and immunoblotted with streptavidin coupled to alkaline phosphatase to detect biotinylated proteins. Asterisk indicates biotinylated *Pf*SET10-TurboID-GFP (~300 kDa). Biotin-treated WT NF54 served as negative control. (**B**) Localization of biotinylated proteins in schizonts. Methanol-fixed schizonts of line *Pf*SET10-TurboID-GFP and WT NF54 were immunolabeled with mouse anti-GFP antibody (green). Biotinylated proteins were detected using fluorophore-conjugated streptavidin (red) and nuclei were highlighted with Hoechst 33342 nuclear stain (blue). Bar; 5 µm. (**C**) Schematic depicting the curation steps during analysis of the *Pf*SET10 interactors.

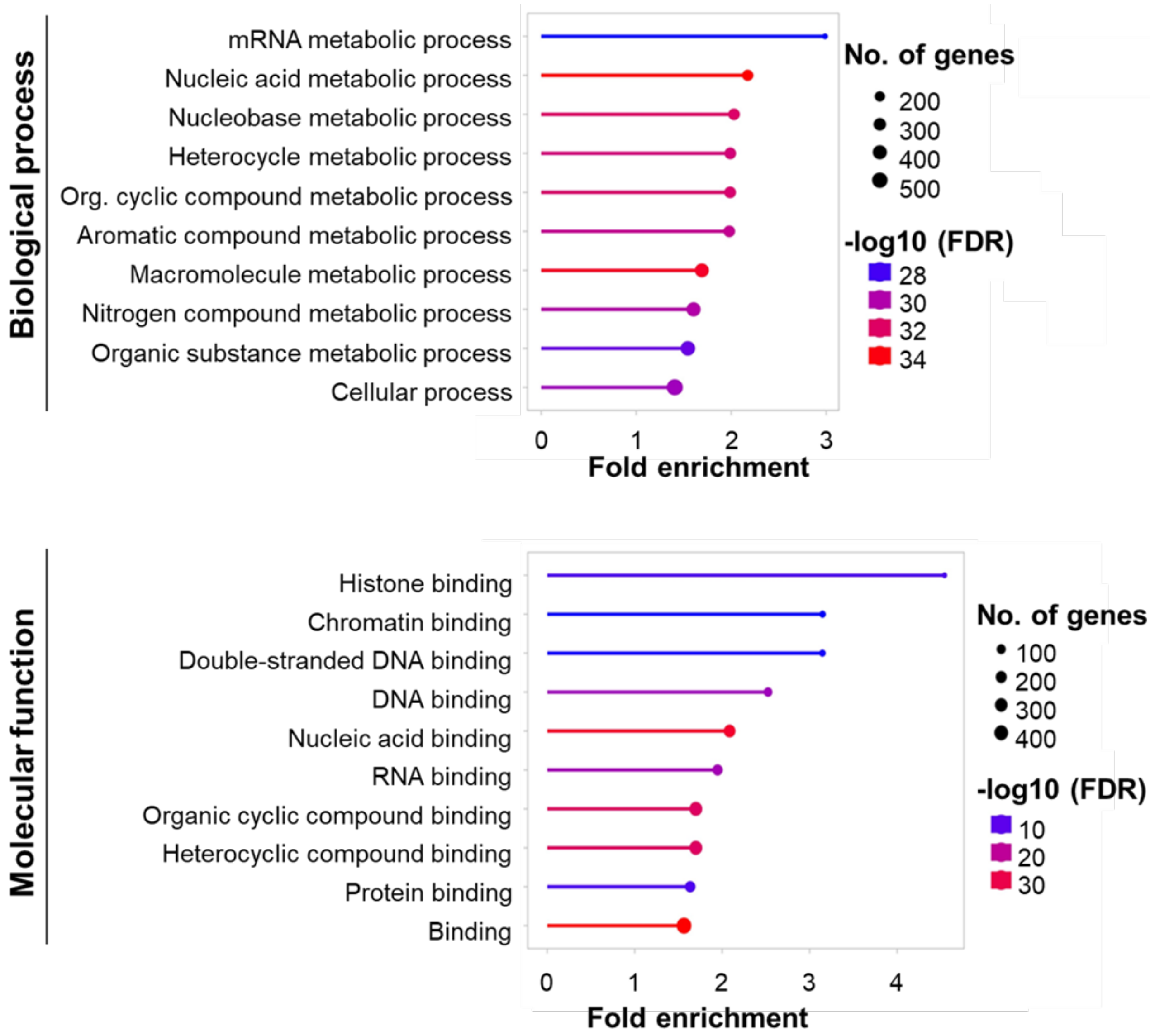

Figure S9. Functional prediction analysis of the *Pf*SET10 interactors. A GO enrichment analysis of the *Pf*SET10 interactors (log2 ratio ≥ 23) was performed using ShinyGO ($p < 0.05$) and the enriched GO terms were based on biological process and molecular function.
