# Supplementary material for: The Plasmodium falciparum histone methyltransferase SET10 participates in a chromatin modulation network crucial for intraerythrocytic development
Source: mSphere. 2024 Oct 24;9(11):e00495-24. doi: 10.1128/msphere.00495-24 (PMC11580448; doi:10.1128/msphere.00495-24)
Supplement: Table S7 — Primer list. [file msphere.00495-24-s0008.pdf]

The *Plasmodium falciparum* histone methyltransferase SET10 participates in a chromatin modulation network crucial for intraerythrocytic development

Jean-Pierre Musabyimana, Sherihan Musa, Janice Manti, Ute Distler, Stefan Tenzer, Che Julius Ngwa, and Gabriele Pradel

Table S7. List of primers for the generation and verification of the *Pf*SET10 HA-KD and *Pf*SET10-TurboID-GFP lines.

**Function**	**Name**	**Sequence 5‘-3‘**
**Primers for the generation and verification of the *Pf*SET10-HA-KD line**		
Cloning primers	*Pf*SET10 pSLI-HA-*glmS* SacII FP	agatctCCGCGGTGGGAAATTACGAATGTCAGAA
	*Pf*SET10 pSLI-HA-*glmS* XhoI RP	agatctCTCGAGACTTGTAGACATAGTTCTTTTT CTTGTTTTTTTA
Vector integration primers	5’ Int *Pf*SET10 pSLI-HA-*glmS* (1)	AAAATTATCGGTTTGTTCAAATTGT
	3’ Int *Pf*SET10 pSLI-HA-*glmS* (2)	TTCATCTTGTTTTCCATTTATTTCC
	pSLI-HA-*glmS* FP (3)	GCTTTACACTTTATGCTTCCGGCTCG
	pSLI-HA-*glmS* RP (4)	TGTCTGTTGTGCCCAGTCAT
**Primers for the generation and verification of the *Pf*SET10-TurboID-GFP line**		
Cloning primers	*Pf*SET10 pSLI-TurboID-GFP NotI FP	tcctccGCGGCCGCAGGTGAAGAAGAAGTGGGAAA
	*Pf*SET10 pSLI-TurboID-GFP SpeI RP	ctttactACTAGTACTTGTAGACATAGTTCTTTTTCTTGT

Vector integration primers	5’ Int *Pf*SET10 pSLI-TurboID-GFP (1)	AAAATTATCGGTTTGTTCAAATTGT
	3’ Int *Pf*SET10 pSLI-TurboID-GFP (2)	TTCATCTTGTTTTCCATTTATTTCC
	pSLI-HA-*glmS* FP (3)	GCTTTACACTTTATGCTTCCGGCTCG
	pSLI-TurboID GFP RP (4)	CAAGTGTTGGCCATGGAA

FP, forward primer; RP, reverse primer. Underlined, sequences of restriction sites.
